# Supplementary material for: Chemical imaging delineates Aβ plaque polymorphism across the Alzheimer’s disease spectrum
Source: Nat Commun. 2025 Apr 24;16:3889. doi: 10.1038/s41467-025-59085-7 (PMC12022071; doi:10.1038/s41467-025-59085-7)
Supplement: Supplementary file 1 — Supplementary Information [file 41467_2025_59085_MOESM1_ESM.pdf]

**Supporting Information**

# Chemical imaging delineates Aβ plaque polymorphism across the Alzheimer's disease spectrum

Srinivas Koutarapu[1#], Junyue Ge[1#], Maciej Dulewicz[1#], Meera Srikrishna [1,2], Alicja Szadziewska [1], Jack Wood [1,3], Kaj Blennow[1,4,5,6], Henrik Zetterberg[1,4,7-10], Wojciech Michno[1,11], Natalie S Ryan [3,12], Tammaryn Lashley[13], Jeffrey Savas[14], Michael Schöll [1,2,3,12,] and Jörg Hanrieder[1,3,4,12, 15*]

1) Department of Psychiatry and Neurochemistry, Sahlgrenska Academy, University of Gothenburg, Mölndal, Sweden

2) Wallenberg Centre for Molecular and Translational Medicine, University of Gothenburg, Gothenburg, Sweden

3) Department of Neuroscience, Physiology and Pharmacology, University College London, London, UK

4) Clinical Neurochemistry Laboratory, Sahlgrenska University Hospital, Mölndal, Sweden

5) Paris Brain Institute, ICM, Pitié-Salpêtrière Hospital, Sorbonne University, Paris, France

6) Neurodegenerative Disorder Research Center, Division of Life Sciences and Medicine, and Department of Neurology, Institute on Aging and Brain Disorders, University of Science and Technology of China and First Affiliated Hospital of USTC, Hefei, P.R. China

7) Department of Neurodegenerative Disease, Institute of Neurology, University College London, London, United Kingdom

8) UK Dementia Research Institute, University College London, London, United Kingdom

9) Hong Kong Centre for Neurodegenerative Diseases, Hong Kong, China

10) Wisconsin Alzheimer's Disease Research Center, University of Wisconsin School of Medicine and Public Health, University of Wisconsin-Madison, Madison, WI, USA

11) Department of Public Health and Caring Sciences, Uppsala University, Uppsala, Sweden

12) Dementia Research Centre, UCL Queen Square Institute of Neurology, University College London, London, United Kingdom

13) Queen Square Brain Bank for Neurological Disorders, Department of Clinical and Movement Neurosciences, UCL Queen Square Institute of Neurology, University College London, London, United Kingdom

14) Ken and Ruth Davee Department of Neurology, Northwestern University Feinberg School of Medicine, Chicago, IL 60611, USA

15) Department of Neuropsychiatry, Sahlgrenska University Hospital, Gothenburg, Sweden

**Contact:**

*Jörg Hanrieder, PhD

Department of Psychiatry and Neurochemistry, Sahlgrenska Academy, University of Gothenburg, Mölndal, Sweden

jh@gu.se; Phone +46-31-3732344

**Content**

SI Table 1+2

SI Figures S1-17

**SI Table S1: Masses of the detected Aβ isoforms.**

| Peptide | Peptide Sequence | Predicted monoisotopic mass $[M+H]^+$ (m/z) | Measured monoisotopic mass in Reflectron mode $[M+H]^+$ (m/z) | Predicted average mass $[M+H]^+$ (m/z) | Measured average mass in Linear mode $[M+H]^+$ (m/z) |
|---|---|---|---|---|---|
| Aβ11pE-40 | pEVHHQKLVFFAEDVGSNKGAIIGLMVGGVV | 3132.68 | Not detected | 3134.7 | 3134.9 |
| Aβ11pE-42 | pEVHHQKLVFFAEDVGSNKGAIIGLMVGGVVIA | 3316.78 | 3316.78 | 3318.9 | 3319.1 |
| Aβ11-42 | EVHHQKLVFFAEDVGSNKGAIIGLMVGGVVIA | 3334.80 | Not detected | 3336.9 | 3336.9 |
| Aβ9-40 | GYEVHHQKLVFFAEDVGSNKGAIIGLMVGGVV | 3370.76 | Not detected | 3372.9 | 3373.2 |
| Aβ8-40 | SGYEVHHQKLVFFAEDVGSNKGAIIGLMVGGVV | 3457.79 | 3457.79 | 3460.0 | 3459.7 |
| Aβ7-40 | DSGYEVHHQKLVFFAEDVGSNKGAIIGLMVGGVV | 3572.82 | 3572.82 | 3575.1 | 3574.9 |
| Aβ4-40 | FRHDSGYEVHHQKLVFFAEDVGSNKGAIIGLMVGGVV | 4013.05 | 4013.05 | 4015.6 | 4015.8 |
| Aβ5-42 | RHDSGYEVHHQKLVFFAEDVGSNKGAIIGLMVGGVVIA | 4050.10 | 4050.08 | 4052.7 | 4053.0 |
| Aβ3pE-40 | pEFRHDSGYEVHHQKLVFFAEDVGSNKGAIIGLMVGGVV | 4124.09 | 4124.09 | 4126.7 | 4126.9 |
| Aβ4-42 | FRHDSGYEVHHQKLVFFAEDVGSNKGAIIGLMVGGVVIA | 4197.17 | 4197.19 | 4199.9 | 4200.4 |
| Aβ3pE-42 | pEFRHDSGYEVHHQKLVFFAEDVGSNKGAIIGLMVGGVVIA | 4308.21 | 4308.20 | 4311.0 | 4311.2 |
| Aβ1-40 | DAEFRHDSGYEVHHQKLVFFAEDVGSNKGAIIGLMVGGVV | 4328.16 | 4328.16 | 4330.9 | 4330.8 |
| Aβ2-42 | AEFRHDSGYEVHHQKLVFFAEDVGSNKGAIIGLMVGGVVIA | 4397.25 | 4397.26 | 4400.1 | 4400.1 |
| Aβ1-42 | DAEFRHDSGYEVHHQKLVFFAEDVGSNKGAIIGLMVGGVVIA | 4512.28 | 4512.27 | 4515.2 | 4515.6 |
| Aβ1-42ox | DAEFRHDSGYEVHHQKLVFFAEDVGSNKGAIIGLMVGGVVIA +oxidation | 4528.27 | Not detected | 4531.2 | 4531.5 |

**Table S2. Misclassified plaque images and their respective true and predicted classes.**

| Misclassified Images | True Class | Predicted Class |
|---|---|---|
| 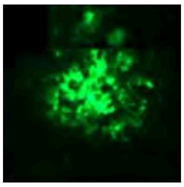 | Coarse Grained Plaque<br>p = 0.38 | Cored Plaque<br>p = 0.42 |
| 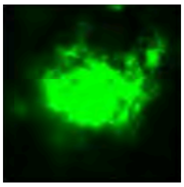 | Cored Plaque<br>p = 0.47 | Coarse Grained Plaque<br>p = 0.51 |
| 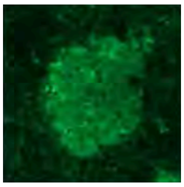 | Diffused Plaque<br>p = 0.48 | Cored Plaque<br>p = 0.51 |

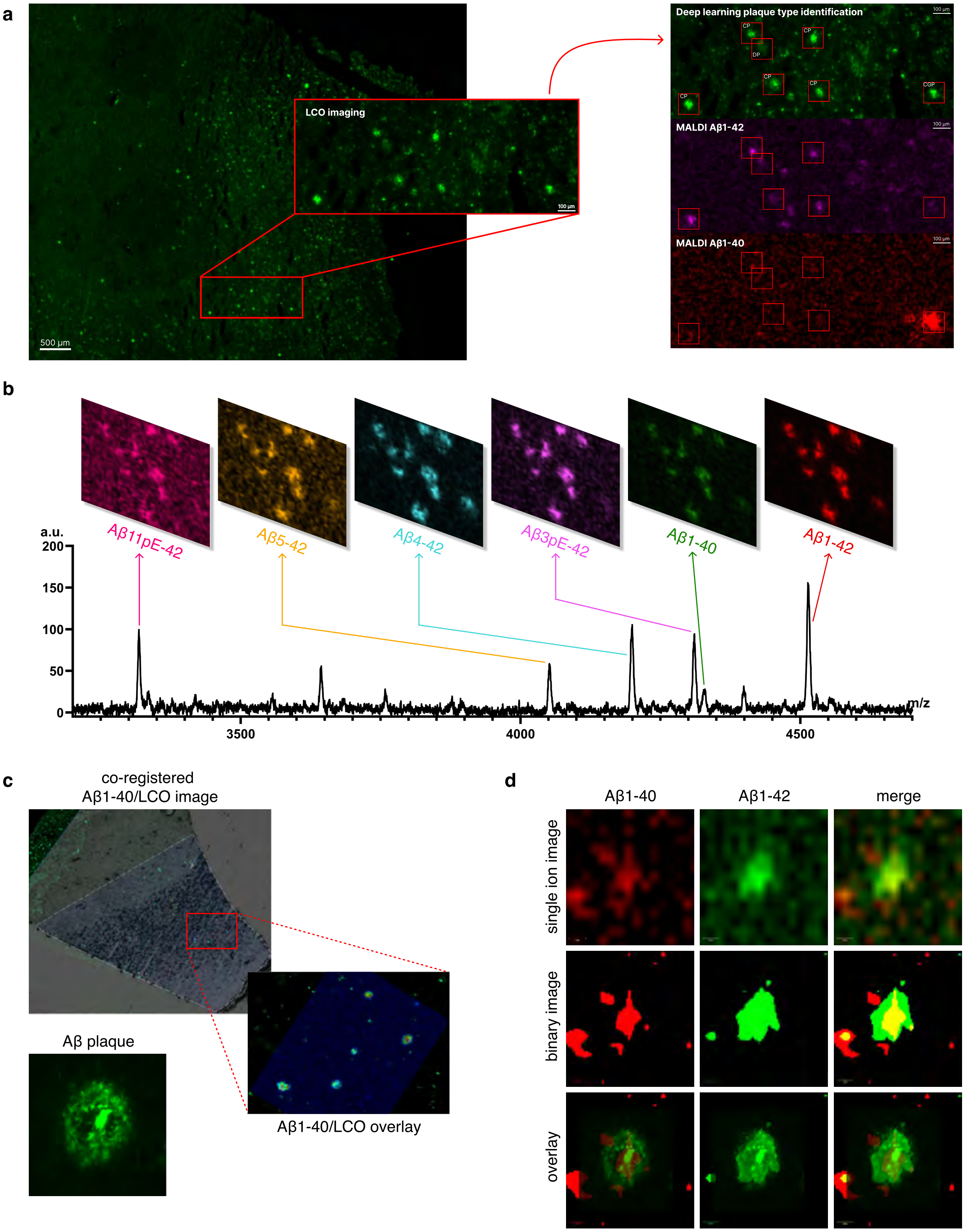


**Figure S1. Technical overview of LCO and MALDI image alignment.**

**a** LCO and MALDI Imaging with Deep Learning plaque type identification. Tissue morphology and plaque distribution showing plaque types polymorphisms recognized by Deep Learning. Corresponding MALDI imaging for Aβ1-42 and Aβ1-40. Rectangles highlight identified plaques by the deep learning algorithm. **b** Mass spectrometry data of different Aβ species: The mass spectrometry spectrum of the analyzed tissue section reveals the presence of various Aβ species in the sample. Individual insets show the ion images for Aβ11pE-42, Aβ5-42, Aβ4-42, Aβ3pE-42, Aβ1-40 and Aβ1-42. **c** Co-registered Aβ1-40/LCO Image. Overlay of acquired fluorescent microscopy image with MALDI data, facilitating the visualization of the spatial correspondence between the LCO signal and Aβ1-40 distribution. **d** Comparison of Aβ1-42 and Aβ1-40 distribution. Single ion and binary images for Aβ1-42 and Aβ1-40. Overlays of the binary images illustrate the co-localization and spatial relationship between Aβ1-42 and Aβ1-40 within the cored plaque.

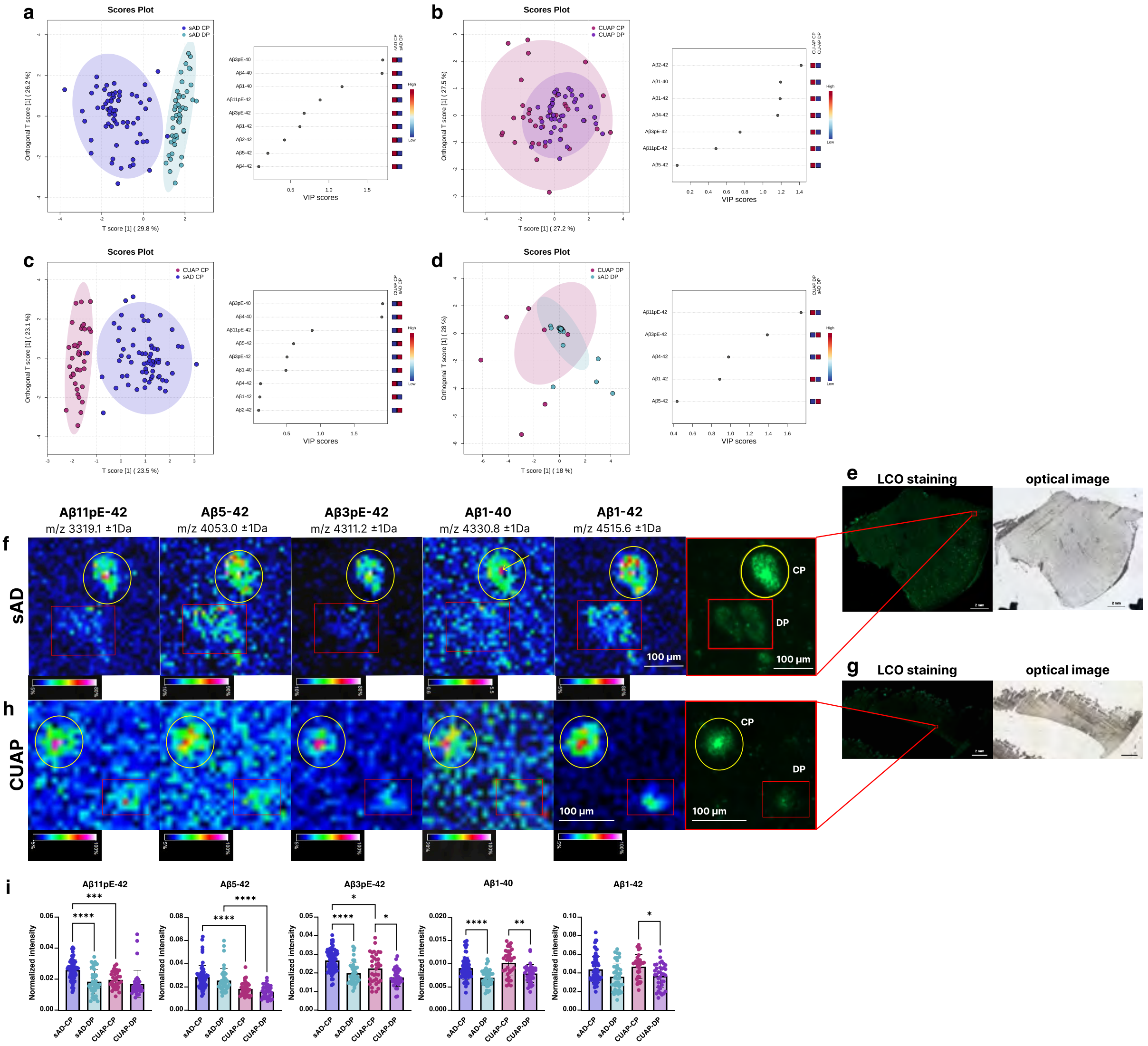


**Figure S2. Multivariate analysis of plaque spectral data of diffuse and cored plaques in sporadic AD and non-demented, cognitively unimpaired amyloid positive.**

**a** OPLS-DA score plots comparing diffuse and cored plaques in sporadic Alzheimer's disease (sAD) and Variable importance in Projection (VIP) scores indicating peptides which separates diffuse plaques from cored plaques in sAD cases. (OPLS model characteristics: R2X- 0.298; R2Y- 0.716; Q2- 0.707). **b** OPLS-DA score plots and VIP scores in cored and diffuse plaques in cognitively unimpaired amyloid positive (CUAP) (OPLS model characteristics: R2X- 0.0635; R2Y- 0.437; Q2- 0.277) **c** OPLS-DA score plots and VIP scores in cored plaques in sAD and CUAP (OPLS model characteristics: R2X- 0.147; R2Y- 0.357; Q2- 0.338) **d** OPLS-DA score plots and VIP scores in diffuse plaques in sAD and CUAP (OPLS model characteristics: R2X- 0.18; R2Y- 0.0638; Q2- -0.0389) **e** LCO microscopy overview of tissue cryosections outlining both diffuse and cored plaques in sAD. **f** Representative MALDI MSI of statistically significant Aβ peptides obtained from one sAD patient. **g** LCO microscopy overview of tissue cryosections outlining both diffuse and cored plaques in CUAP. **h** Representative MALDI MSI of statistically significant Aβ peptides obtained from one CUAP patient. Intensity scale: rel. intensity in %. Scalebar: 100um **i** Corresponding bar plots showing univariate comparison of single ion intensities with respective plaque types. Plots indicate single plaque values mean±SD. Number of patients: n=12 sAD and n=5 CUAP; number of plaques per patient and plaque type N=5-15. Univariate analysis was performed using Kruskall-Wallis test with two-sided Dunn's correction: Aβ11pE-42: sAD-CP vs. sAD-DP $p < 0.0001$, sAD-CP vs. CUAP-CP $p = 0.0008$; Aβ5-42: sAD-CP vs. CUAP-CP $p < 0.0001$, sAD-DP vs. CUAP-DP $p < 0.0001$; Aβ3pE-42: sAD-CP vs. sAD-DP $p < 0.0001$, sAD-CP vs. CUAP-CP $p = 0.0185$, CUAP-CP vs. CUAP-DP $p = 0.0232$; Aβ1-40: sAD-CP vs. sAD-DP $p < 0.0001$, CUAP-CP vs. CUAP-DP $p < 0.01$; Aβ1-42: CUAP-CP vs. CUAP-DP $p < 0.05$ (*p<0.05, **p<0.01, ***p<0.001, ****p<0.0001).

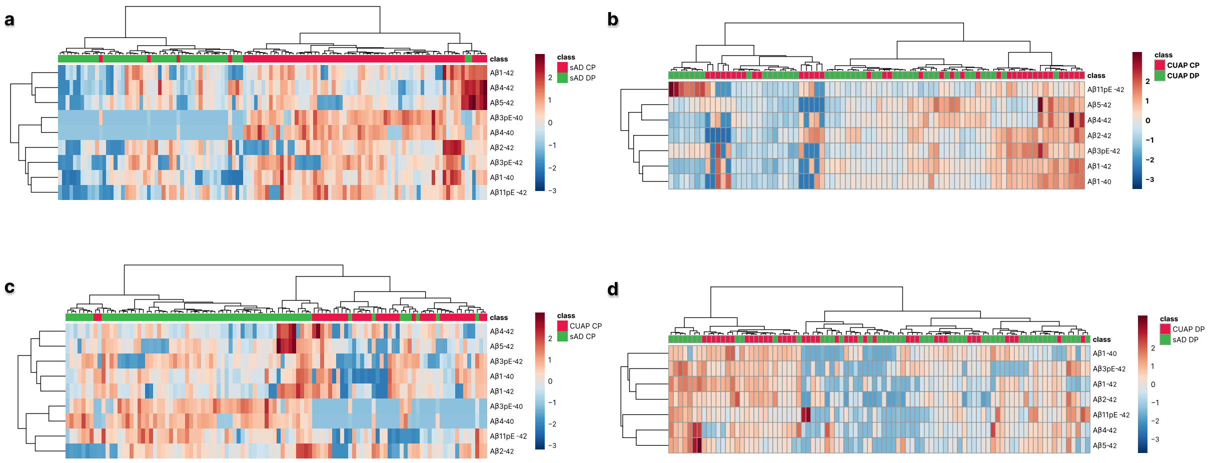


**Figure S3. Cluster analysis of plaque spectral data for diffuse and cored plaques in CUAP and sAD.**

Clustered heat maps of averaged peptide intensities illustrating characteristic differences in between plaque populations driven by x-40 and x-42 species. **a-b** Comparison of CP and DP in sAD shows strong separation in two main clusters, while this was not prominent for CP vs DP in CUAP. **c-d** A strong separation was observed for CP across sAD and CUAP, while diffuse plaques in between sAD and CUAP appeared quite similar.

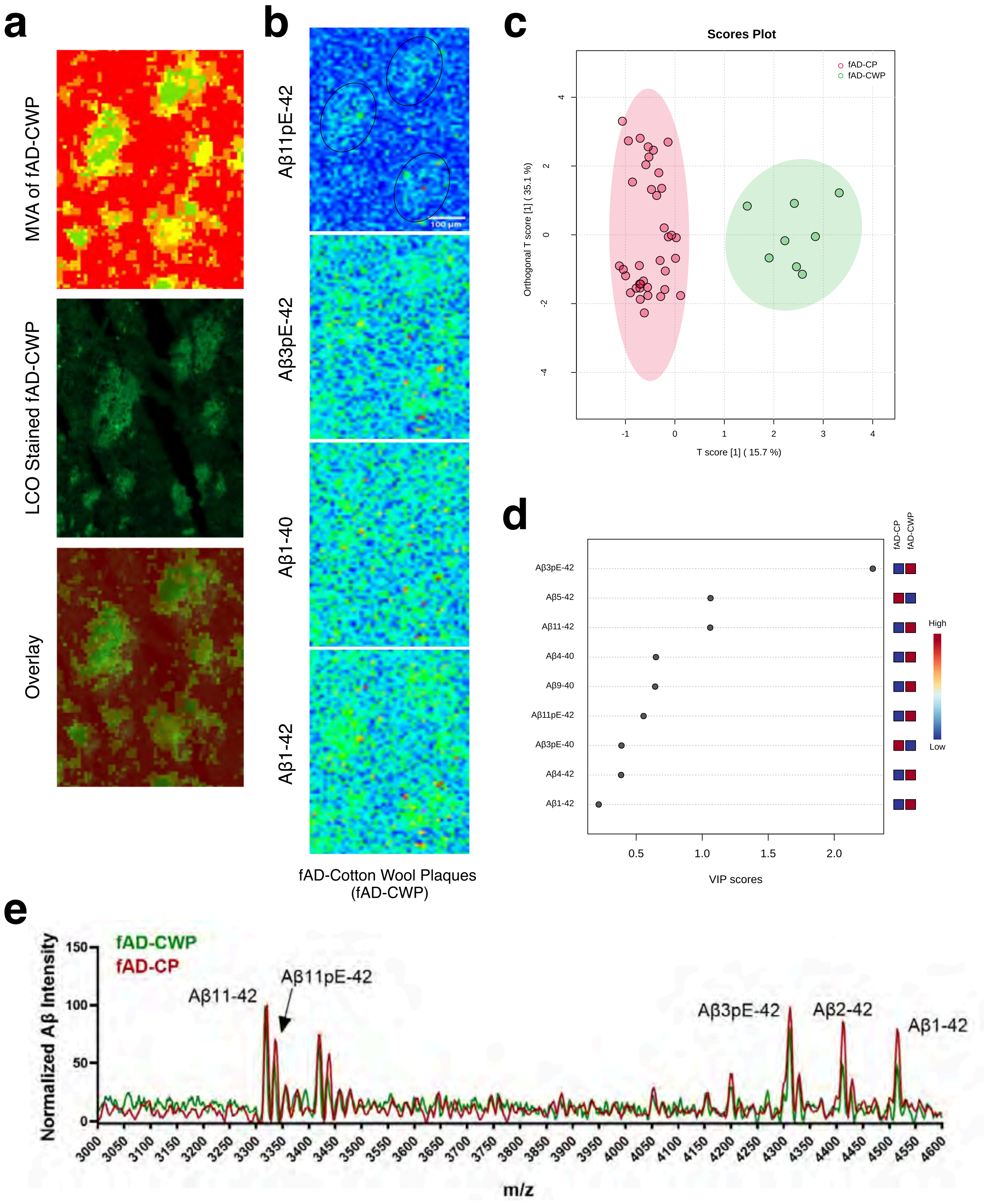


**Figure S4. Plaque Aβ pattern of cotton wool plaques (CWP) observed in one familial Alzheimer's disease (fAD) patient.**

**a** Cluster analysis based image segmentation analysis map of MALDI MSI data from fAD brain tissue. Overlay of segmentation map and LCO imaging identifies CWP associated MSI profiles. **b** Single ion images for Aβ11pE-42, Aβ3pE-42, Aβ1-40, Aβ1-42. **c** OPLS-DA for comparing cotton wool plaques (CWP) with cored plaques (CP) showing strong separation. (OPLS model characteristics: R2X-0.157; R2Y-0.737; Q2-0.604) **d** Variable importance in Projection (VIP) scores indicating peptides which separate cotton wool plaques from cored plaques. **e** Average mass spectra of CWP (N=9) and CP (N=46) detected within one fAD patient. The spectra are basepeak normalized (11pE-42) to illustrate relatively higher degrees of pyroglutamated species as compared to Aβ1-42 (m/z 4515) in cotton wool plaques (CWP) as compared to cored plaques (CP). Intensity scale b: rel. intensity in %. Scalebar b: 100um

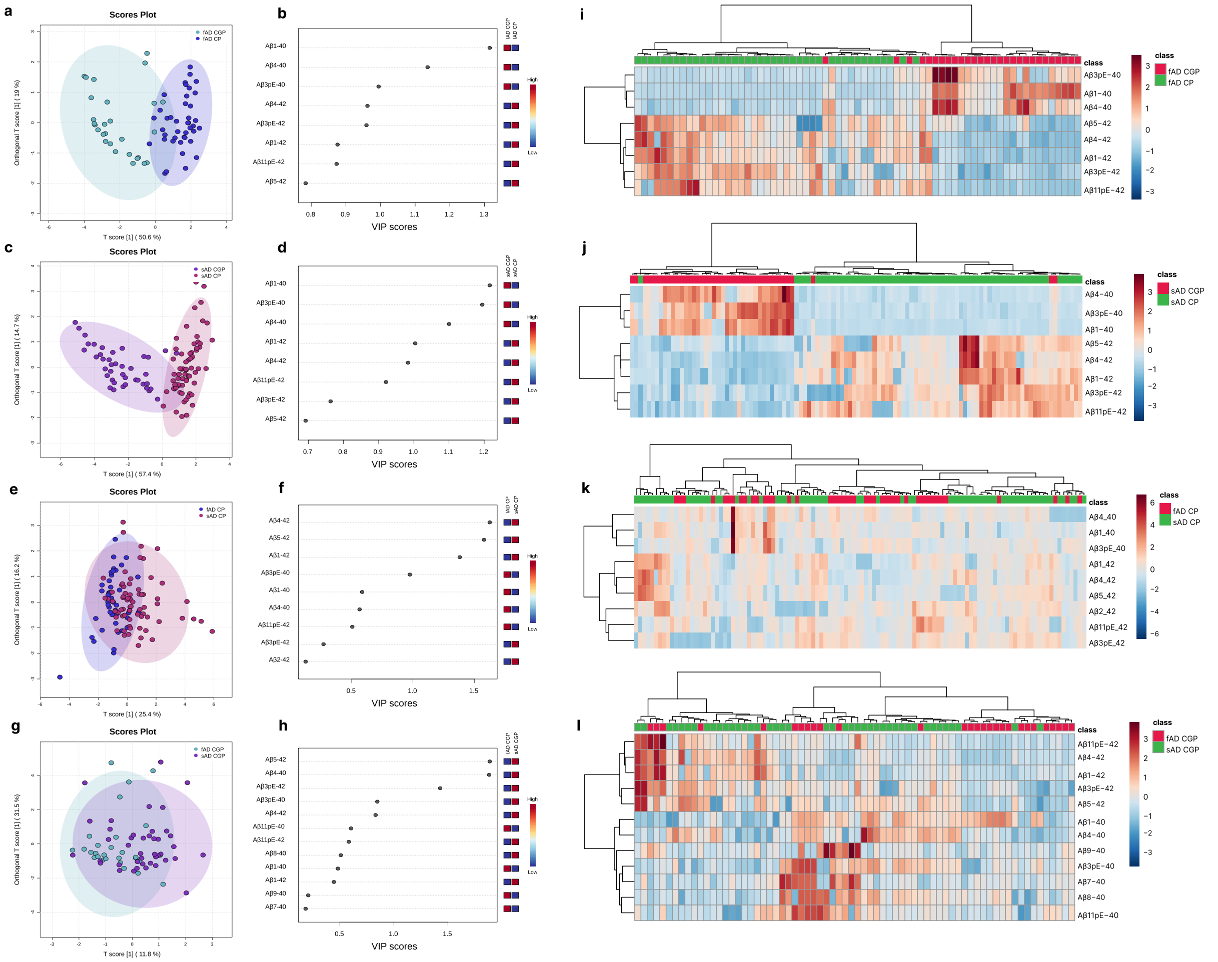


**Figure S5. Multivariate analysis of Aβ patterns in cored- and coarse grain plaques in sporadic Alzheimer's disease (sAD) and familial Alzheimer's disease (fAD).**

**a** OPLS-DA score plots and **b** Variable importance in Projection (VIP) scores comparing fAD coarse-grained plaques with fAD cored plaques (OPLS model characteristics: R2X-0.506; R2Y-0.655; Q2-0.649). **c** OPLS-DA score plots and **d** Variable importance in Projection (VIP) scores sAD coarse grain plaques with sAD cored plaques (OPLS model characteristics: R2X-0.574; R2Y-0.749; Q2-0.744). **e** OPLS-DA score plots and **f** Variable importance in Projection (VIP) scores comparing sAD cored plaques with fAD cored plaques (OPLS model characteristics: R2X-0.254; R2Y-0.263; Q2-0.239). **g** OPLS-DA score plots and **h** Variable importance in Projection (VIP) scores comparing fAD coarse grain plaques with sAD coarse grain plaques (OPLS model characteristics: R2X-0.118; R2Y-0.104; Q2-0.00769). **i-l** Clustered heat maps of averaged peptide intensities illustrating characteristic differences in between plaque populations. Comparison of CP and CGP in **i** fAD and **j** sAD shows strong separation in two main clusters driven by x-40 (CGP) and x-42 species (DP). **k** Cored plaques and **l** coarse grained plaques in between sAD and fAD did not show any separation in the cluster analysis.

## Multivariate

### Correlations

| | Aβ9-40 | Aβ4-40 | Aβ3pE-40 | Aβ1-40 | Aβ11pE-42 | Aβ11-42 | Aβ5-42 | Aβ4-42 | Aβ4-42ox | Aβ3pE-42 | Aβ2-42 | Aβ1-42 | Aβ1-42ox |
|---|---|---|---|---|---|---|---|---|---|---|---|---|---|
| Aβ9-40 | 1,0000 | 0,3534 | 0,6564 | 0,1585 | 0,0011 | 0,4500 | -0,7223 | -0,6623 | -0,2124 | -0,3317 | 0,4308 | -0,7006 | 0,2676 |
| Aβ4-40 | 0,3534 | 1,0000 | 0,2833 | 0,3385 | -0,0880 | 0,2924 | -0,4726 | -0,3925 | -0,2782 | -0,0600 | 0,4256 | -0,3680 | 0,0816 |
| Aβ3pE-40 | 0,6564 | 0,2833 | 1,0000 | 0,3872 | 0,1358 | 0,4124 | -0,7041 | -0,7370 | -0,1672 | -0,1009 | 0,5630 | -0,6650 | 0,3986 |
| Aβ1-40 | 0,1585 | 0,3385 | 0,3872 | 1,0000 | 0,0391 | 0,1057 | -0,4825 | -0,3732 | -0,1796 | 0,0494 | 0,1951 | -0,2515 | 0,0794 |
| Aβ11pE-42 | 0,0011 | -0,0880 | 0,1358 | 0,0391 | 1,0000 | 0,6199 | -0,1975 | -0,4846 | -0,2969 | 0,5430 | -0,0529 | -0,3540 | -0,3034 |
| Aβ11-42 | 0,4500 | 0,2924 | 0,4124 | 0,1057 | 0,6199 | 1,0000 | -0,4273 | -0,5766 | -0,0516 | -0,0670 | 0,0878 | -0,7785 | 0,0446 |
| Aβ5-42 | -0,7223 | -0,4726 | -0,7041 | -0,4825 | -0,1975 | -0,4273 | 1,0000 | 0,8523 | 0,5770 | -0,1111 | -0,6954 | 0,5018 | -0,3238 |
| Aβ4-42 | -0,6623 | -0,3925 | -0,7370 | -0,3732 | -0,4846 | -0,5766 | 0,8523 | 1,0000 | 0,5571 | -0,2941 | -0,5998 | 0,6777 | -0,2572 |
| Aβ4-42ox | -0,2124 | -0,2782 | -0,1672 | -0,1796 | -0,2969 | -0,0516 | 0,5770 | 0,5571 | 1,0000 | -0,6760 | -0,4449 | -0,0275 | 0,2229 |
| Aβ3pE-42 | -0,3317 | -0,0600 | -0,1009 | 0,0494 | 0,5430 | -0,0670 | -0,1111 | -0,2941 | -0,6760 | 1,0000 | 0,1252 | 0,2632 | -0,2972 |
| Aβ2-42 | 0,4308 | 0,4256 | 0,5630 | 0,1951 | -0,0529 | 0,0878 | -0,6954 | -0,5998 | -0,4449 | 0,1252 | 1,0000 | -0,1361 | 0,4879 |
| Aβ1-42 | -0,7006 | -0,3680 | -0,6650 | -0,2515 | -0,3540 | -0,7785 | 0,5018 | 0,6777 | -0,0275 | 0,2632 | -0,1361 | 1,0000 | -0,1757 |
| Aβ1-42ox | 0,2676 | 0,0816 | 0,3986 | 0,0794 | -0,3034 | 0,0446 | -0,3238 | -0,2572 | 0,2229 | -0,2972 | 0,4879 | -0,1757 | 1,0000 |

The correlations are estimated by Row-wise method.

### Correlation Probability

| | Aβ9-40 | Aβ4-40 | Aβ3pE-40 | Aβ1-40 | Aβ11pE-42 | Aβ11-42 | Aβ5-42 | Aβ4-42 | Aβ4-42ox | Aβ3pE-42 | Aβ2-42 | Aβ1-42 | Aβ1-42ox |
|---|---|---|---|---|---|---|---|---|---|---|---|---|---|
| Aβ9-40 | <,0001 | 0,0554 | <,0001 | 0,4028 | 0,9955 | 0,0126 | <,0001 | <,0001 | 0,2597 | 0,0734 | 0,0175 | <,0001 | 0,1529 |
| Aβ4-40 | 0,0554 | <,0001 | 0,1292 | 0,0673 | 0,6439 | 0,1168 | 0,0084 | 0,0319 | 0,1366 | 0,7529 | 0,0190 | 0,0454 | 0,6683 |
| Aβ3pE-40 | <,0001 | 0,1292 | <,0001 | 0,0345 | 0,4743 | 0,0235 | <,0001 | <,0001 | 0,3771 | 0,5957 | 0,0012 | <,0001 | 0,0291 |
| Aβ1-40 | 0,4028 | 0,0673 | 0,0345 | <,0001 | 0,8376 | 0,5781 | 0,0069 | 0,0423 | 0,3423 | 0,7956 | 0,3016 | 0,1801 | 0,6767 |
| Aβ11pE-42 | 0,9955 | 0,6439 | 0,4743 | 0,8376 | <,0001 | 0,0003 | 0,2955 | 0,0066 | 0,1111 | 0,0019 | 0,7814 | 0,0550 | 0,1031 |
| Aβ11-42 | 0,0126 | 0,1168 | 0,0235 | 0,5781 | 0,0003 | <,0001 | 0,0185 | 0,0009 | 0,7866 | 0,7251 | 0,6446 | <,0001 | 0,8151 |
| Aβ5-42 | <,0001 | 0,0084 | <,0001 | 0,0069 | 0,2955 | 0,0185 | <,0001 | <,0001 | 0,0008 | 0,5588 | <,0001 | 0,0047 | 0,0809 |
| Aβ4-42 | <,0001 | 0,0319 | <,0001 | 0,0423 | 0,0066 | 0,0009 | <,0001 | <,0001 | 0,0014 | 0,1147 | 0,0005 | <,0001 | 0,1700 |
| Aβ4-42ox | 0,2597 | 0,1366 | 0,3771 | 0,3423 | 0,1111 | 0,7866 | 0,0008 | 0,0014 | <,0001 | <,0001 | 0,0138 | 0,8853 | 0,2365 |
| Aβ3pE-42 | 0,0734 | 0,7529 | 0,5957 | 0,7956 | 0,0019 | 0,7251 | 0,5588 | 0,1147 | <,0001 | <,0001 | 0,5097 | 0,1600 | 0,1107 |
| Aβ2-42 | 0,0175 | 0,0190 | 0,0012 | 0,3016 | 0,7814 | 0,6446 | <,0001 | 0,0005 | 0,0138 | 0,5097 | <,0001 | 0,4733 | 0,0062 |
| Aβ1-42 | <,0001 | 0,0454 | <,0001 | 0,1801 | 0,0550 | <,0001 | 0,0047 | <,0001 | 0,8853 | 0,1600 | 0,4733 | <,0001 | 0,3530 |
| Aβ1-42ox | 0,1529 | 0,6683 | 0,0291 | 0,6767 | 0,1031 | 0,8151 | 0,0809 | 0,1700 | 0,2365 | 0,1107 | 0,0062 | 0,3530 | <,0001 |

### Scatterplot Matrix

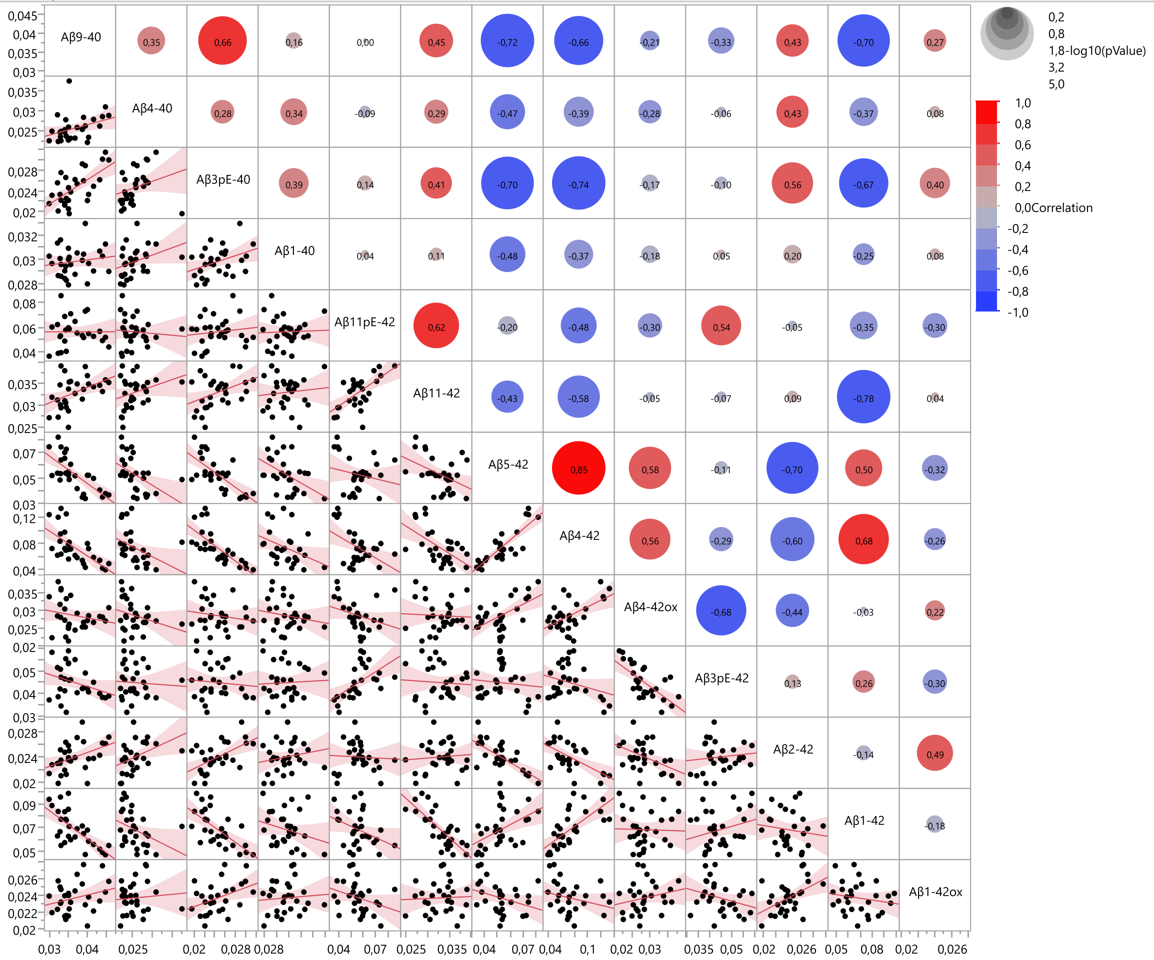


**Figure S6.**

Correlation analysis of amyloid peptides in sAD-Cored Plaques. Correlation matrix of all amyloid peptides indicating both correlation plots, significance (p value) and correlation $R^2$.

## Multivariate

### Correlations

| | Aβ9-40 | Aβ4-40 | Aβ3pE-40 | Aβ1-40 | Aβ11pE-42 | Aβ11-42 | Aβ5-42 | Aβ4-42 | Aβ4-42ox | Aβ3pE-42 | Aβ2-42 | Aβ1-42 | Aβ1-42ox |
|---|---|---|---|---|---|---|---|---|---|---|---|---|---|
| Aβ9-40 | 1,0000 | 0,5357 | 0,4743 | 0,1253 | -0,2066 | 0,0930 | -0,6195 | -0,8162 | -0,3301 | -0,5628 | -0,0538 | -0,8016 | -0,4305 |
| Aβ4-40 | 0,5357 | 1,0000 | 0,9249 | 0,5772 | -0,6142 | -0,4736 | -0,1794 | -0,5711 | 0,1233 | -0,5492 | 0,0502 | -0,6335 | -0,2495 |
| Aβ3pE-40 | 0,4743 | 0,9249 | 1,0000 | 0,6967 | -0,5760 | -0,4294 | -0,1877 | -0,4841 | 0,1562 | -0,5256 | 0,0257 | -0,5418 | -0,2187 |
| Aβ1-40 | 0,1253 | 0,5772 | 0,6967 | 1,0000 | -0,6853 | -0,6022 | 0,2144 | 0,0592 | 0,4164 | 0,0938 | 0,4828 | 0,0768 | 0,3673 |
| Aβ11pE-42 | -0,2066 | -0,6142 | -0,5760 | -0,6853 | 1,0000 | 0,8741 | -0,3413 | 0,0473 | -0,5975 | -0,0147 | -0,6795 | 0,0834 | -0,4220 |
| Aβ11-42 | 0,0930 | -0,4736 | -0,4294 | -0,6022 | 0,8741 | 1,0000 | -0,5210 | -0,1209 | -0,5671 | -0,1892 | -0,5955 | -0,0965 | -0,4050 |
| Aβ5-42 | -0,6195 | -0,1794 | -0,1877 | 0,2144 | -0,3413 | -0,5210 | 1,0000 | 0,6981 | 0,4994 | 0,5898 | 0,4855 | 0,6194 | 0,5158 |
| Aβ4-42 | -0,8162 | -0,5711 | -0,4841 | 0,0592 | 0,0473 | -0,1209 | 0,6981 | 1,0000 | 0,5913 | 0,5845 | 0,2649 | 0,9462 | 0,7211 |
| Aβ4-42ox | -0,3301 | 0,1233 | 0,1562 | 0,4164 | -0,5975 | -0,5671 | 0,4994 | 0,5913 | 1,0000 | 0,0737 | 0,4554 | 0,4622 | 0,7447 |
| Aβ3pE-42 | -0,5628 | -0,5492 | -0,5256 | 0,0938 | -0,0147 | -0,1892 | 0,5898 | 0,5845 | 0,0737 | 1,0000 | 0,4553 | 0,7018 | 0,4776 |
| Aβ2-42 | -0,0538 | 0,0502 | 0,0257 | 0,4828 | -0,6795 | -0,5955 | 0,4855 | 0,2649 | 0,4554 | 0,4553 | 1,0000 | 0,3452 | 0,6650 |
| Aβ1-42 | -0,8016 | -0,6335 | -0,5418 | 0,0768 | 0,0834 | -0,0965 | 0,6194 | 0,9462 | 0,4622 | 0,7018 | 0,3452 | 1,0000 | 0,7519 |
| Aβ1-42ox | -0,4305 | -0,2495 | -0,2187 | 0,3673 | -0,4220 | -0,4050 | 0,5158 | 0,7211 | 0,7447 | 0,4776 | 0,6650 | 0,7519 | 1,0000 |

The correlations are estimated by Row-wise method.

### Correlation Probability

| | Aβ9-40 | Aβ4-40 | Aβ3pE-40 | Aβ1-40 | Aβ11pE-42 | Aβ11-42 | Aβ5-42 | Aβ4-42 | Aβ4-42ox | Aβ3pE-42 | Aβ2-42 | Aβ1-42 | Aβ1-42ox |
|---|---|---|---|---|---|---|---|---|---|---|---|---|---|
| Aβ9-40 | <,0001 | 0,0001 | 0,0009 | 0,4067 | 0,1683 | 0,5387 | <,0001 | <,0001 | 0,0251 | <,0001 | 0,7227 | <,0001 | 0,0028 |
| Aβ4-40 | 0,0001 | <,0001 | <,0001 | <,0001 | <,0001 | 0,0009 | 0,2330 | <,0001 | 0,4142 | <,0001 | 0,7402 | <,0001 | 0,0944 |
| Aβ3pE-40 | 0,0009 | <,0001 | <,0001 | <,0001 | <,0001 | 0,0029 | 0,2116 | 0,0007 | 0,2998 | 0,0002 | 0,8652 | 0,0001 | 0,1442 |
| Aβ1-40 | 0,4067 | <,0001 | <,0001 | <,0001 | <,0001 | <,0001 | 0,1526 | 0,6957 | 0,0040 | 0,5353 | 0,0007 | 0,6120 | 0,0120 |
| Aβ11pE-42 | 0,1683 | <,0001 | <,0001 | <,0001 | <,0001 | <,0001 | 0,0203 | 0,7551 | <,0001 | 0,9229 | <,0001 | 0,5814 | 0,0035 |
| Aβ11-42 | 0,5387 | 0,0009 | 0,0029 | <,0001 | <,0001 | <,0001 | 0,0002 | 0,4237 | <,0001 | 0,2080 | <,0001 | 0,5237 | 0,0052 |
| Aβ5-42 | <,0001 | 0,2330 | 0,2116 | 0,1526 | 0,0203 | 0,0002 | <,0001 | <,0001 | 0,0004 | <,0001 | 0,0006 | <,0001 | 0,0002 |
| Aβ4-42 | <,0001 | <,0001 | 0,0007 | 0,6957 | 0,7551 | 0,4237 | <,0001 | <,0001 | <,0001 | <,0001 | 0,0752 | <,0001 | <,0001 |
| Aβ4-42ox | 0,0251 | 0,4142 | 0,2998 | 0,0040 | <,0001 | <,0001 | 0,0004 | <,0001 | <,0001 | 0,6264 | 0,0015 | 0,0012 | <,0001 |
| Aβ3pE-42 | <,0001 | <,0001 | 0,0002 | 0,5353 | 0,9229 | 0,2080 | <,0001 | <,0001 | 0,6264 | <,0001 | 0,0015 | <,0001 | 0,0008 |
| Aβ2-42 | 0,7227 | 0,7402 | 0,8652 | 0,0007 | <,0001 | <,0001 | 0,0006 | 0,0752 | 0,0015 | 0,0015 | <,0001 | 0,0188 | <,0001 |
| Aβ1-42 | <,0001 | <,0001 | 0,0001 | 0,6120 | 0,5814 | 0,5237 | <,0001 | <,0001 | 0,0012 | <,0001 | 0,0188 | <,0001 | <,0001 |
| Aβ1-42ox | 0,0028 | 0,0944 | 0,1442 | 0,0120 | 0,0035 | 0,0052 | 0,0002 | <,0001 | <,0001 | 0,0008 | <,0001 | <,0001 | <,0001 |

### Scatterplot Matrix

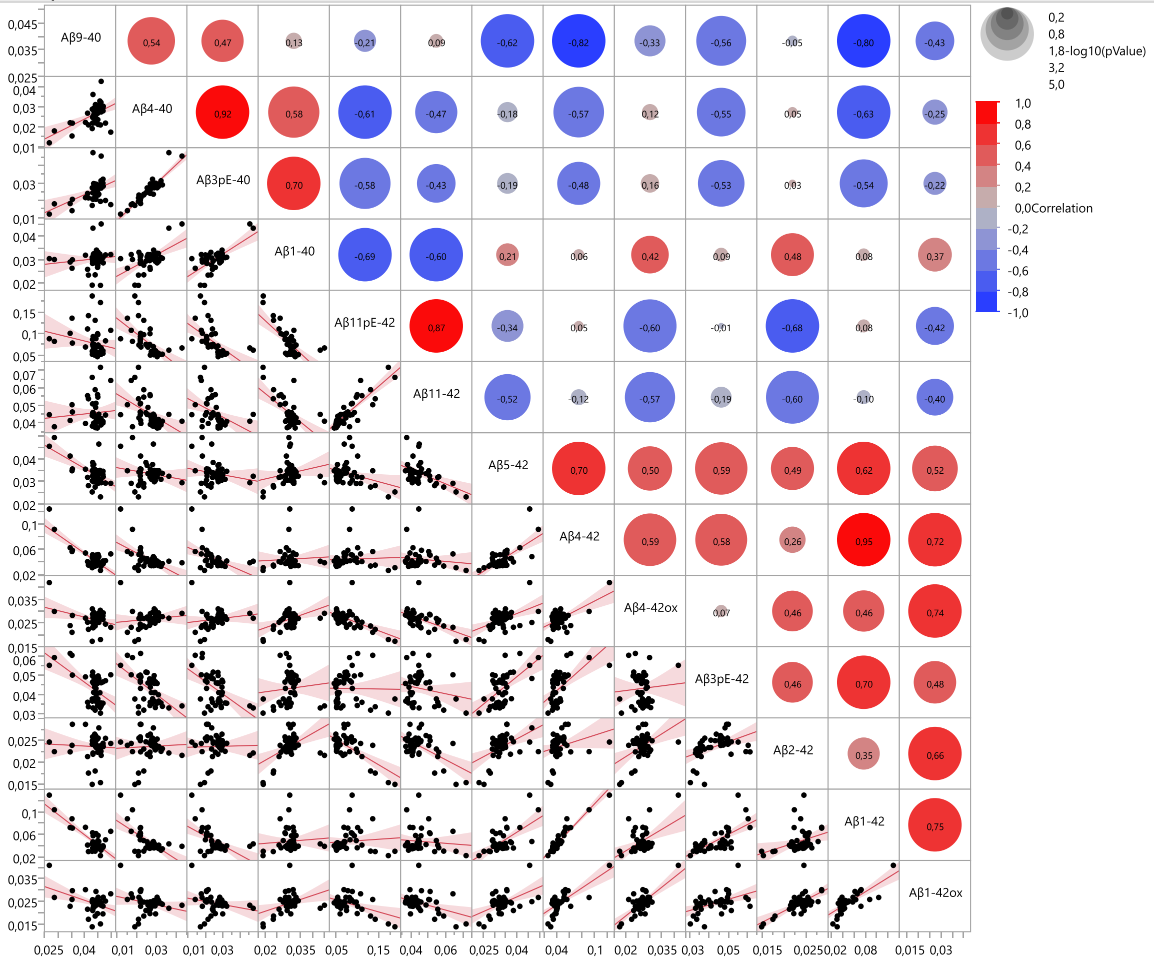


**Figure S7.**

Correlation analysis of amyloid peptides in fAD-Cored Plaques. Correlation matrix of all amyloid peptides indicating both correlation plots, significance (p value) and correlation $R^2$.

## Multivariate

### Correlations

| | Aβ11pE_40 | Aβ9-40 | Aβ8-40 | Aβ7-40 | Aβ5-40 | Aβ4-40 | Aβ3pE-40 | Aβ3-40 | Aβ2-40 | Aβ1-40 | Aβ11pE-42 | Aβ11-42 | Aβ5-42 | Aβ4-42 | Aβ3pE-42 | Aβ1-42 |
|---|---|---|---|---|---|---|---|---|---|---|---|---|---|---|---|---|
| Aβ11pE_40 | 1,0000 | 0,9566 | 0,2861 | 0,9408 | -0,4047 | -0,7892 | -0,6240 | -0,5966 | 0,0343 | -0,8630 | 0,8143 | 0,9481 | 0,5883 | 0,7831 | 0,6973 | 0,7591 |
| Aβ9-40 | 0,9566 | 1,0000 | 0,2620 | 0,9403 | -0,4526 | -0,7645 | -0,6609 | -0,5635 | 0,0415 | -0,8671 | 0,8096 | 0,9631 | 0,5874 | 0,8123 | 0,6737 | 0,7827 |
| Aβ8-40 | 0,2861 | 0,2620 | 1,0000 | 0,3410 | -0,0917 | -0,7134 | 0,4783 | 0,1881 | 0,1777 | -0,0118 | 0,1093 | 0,1649 | -0,4083 | -0,1250 | -0,2078 | -0,1600 |
| Aβ7-40 | 0,9408 | 0,9403 | 0,3410 | 1,0000 | -0,4146 | -0,8004 | -0,5452 | -0,4494 | -0,0675 | -0,8301 | 0,7442 | 0,9325 | 0,4976 | 0,7377 | 0,6262 | 0,7184 |
| Aβ5-40 | -0,4047 | -0,4526 | -0,0917 | -0,4146 | 1,0000 | 0,5417 | 0,3227 | 0,3124 | -0,4324 | 0,2031 | -0,3180 | -0,4681 | -0,2479 | -0,3509 | -0,4164 | -0,4044 |
| Aβ4-40 | -0,7892 | -0,7645 | -0,7134 | -0,8004 | 0,5417 | 1,0000 | 0,1620 | 0,3810 | -0,2488 | 0,5230 | -0,6258 | -0,7229 | -0,1756 | -0,4715 | -0,4292 | -0,4674 |
| Aβ3pE-40 | -0,6240 | -0,6609 | 0,4783 | -0,5452 | 0,3227 | 0,1620 | 1,0000 | 0,7764 | 0,0586 | 0,7914 | -0,7031 | -0,7169 | -0,9110 | -0,8924 | -0,8574 | -0,8824 |
| Aβ3-40 | -0,5966 | -0,5635 | 0,1881 | -0,4494 | 0,3124 | 0,3810 | 0,7764 | 1,0000 | -0,1391 | 0,6605 | -0,7791 | -0,5969 | -0,7961 | -0,7954 | -0,8763 | -0,8077 |
| Aβ2-40 | 0,0343 | 0,0415 | 0,1777 | -0,0675 | -0,4324 | -0,2488 | 0,0586 | -0,1391 | 1,0000 | 0,2693 | -0,2178 | -0,0815 | -0,0685 | -0,1455 | -0,0898 | -0,1621 |
| Aβ1-40 | -0,8630 | -0,8671 | -0,0118 | -0,8301 | 0,2031 | 0,5230 | 0,7914 | 0,6605 | 0,2693 | 1,0000 | -0,9024 | -0,9288 | -0,8163 | -0,9417 | -0,8334 | -0,9207 |
| Aβ11pE-42 | 0,8143 | 0,8096 | 0,1093 | 0,7442 | -0,3180 | -0,6258 | -0,7031 | -0,7791 | -0,2178 | -0,9024 | 1,0000 | 0,8783 | 0,7169 | 0,8993 | 0,8559 | 0,9028 |
| Aβ11-42 | 0,9481 | 0,9631 | 0,1649 | 0,9325 | -0,4681 | -0,7229 | -0,7169 | -0,5969 | -0,0815 | -0,9288 | 0,8783 | 1,0000 | 0,6770 | 0,8756 | 0,7702 | 0,8645 |
| Aβ5-42 | 0,5883 | 0,5874 | -0,4083 | 0,4976 | -0,2479 | -0,1756 | -0,9110 | -0,7961 | -0,0685 | -0,8163 | 0,7169 | 0,6770 | 1,0000 | 0,8890 | 0,8954 | 0,8992 |
| Aβ4-42 | 0,7831 | 0,8123 | -0,1250 | 0,7377 | -0,3509 | -0,4715 | -0,8924 | -0,7954 | -0,1455 | -0,9417 | 0,8993 | 0,8756 | 0,8890 | 1,0000 | 0,9269 | 0,9866 |
| Aβ3pE-42 | 0,6973 | 0,6737 | -0,2078 | 0,6262 | -0,4164 | -0,4292 | -0,8574 | -0,8763 | -0,0898 | -0,8334 | 0,8559 | 0,7702 | 0,8954 | 0,9269 | 1,0000 | 0,9510 |
| Aβ1-42 | 0,7591 | 0,7827 | -0,1600 | 0,7184 | -0,4044 | -0,4674 | -0,8824 | -0,8077 | -0,1621 | -0,9207 | 0,9028 | 0,8645 | 0,8992 | 0,9866 | 0,9510 | 1,0000 |

The correlations are estimated by Row-wise method.

### Correlation Probability

| | Aβ11pE_40 | Aβ9-40 | Aβ8-40 | Aβ7-40 | Aβ5-40 | Aβ4-40 | Aβ3pE-40 | Aβ3-40 | Aβ2-40 | Aβ1-40 | Aβ11pE-42 | Aβ11-42 | Aβ5-42 | Aβ4-42 | Aβ3pE-42 | Aβ1-42 |
|---|---|---|---|---|---|---|---|---|---|---|---|---|---|---|---|---|
| Aβ11pE_40 | <,0001 | <,0001 | 0,1754 | <,0001 | 0,0498 | <,0001 | 0,0011 | 0,0021 | 0,8735 | <,0001 | <,0001 | <,0001 | 0,0025 | <,0001 | 0,0002 | <,0001 |
| Aβ9-40 | <,0001 | <,0001 | 0,2162 | <,0001 | 0,0264 | <,0001 | 0,0004 | 0,0041 | 0,8473 | <,0001 | <,0001 | <,0001 | 0,0025 | <,0001 | 0,0003 | <,0001 |
| Aβ8-40 | 0,1754 | 0,2162 | <,0001 | 0,1030 | 0,6700 | <,0001 | 0,0181 | 0,3789 | 0,4061 | 0,9564 | 0,6111 | 0,4412 | 0,0476 | 0,5605 | 0,3299 | 0,4552 |
| Aβ7-40 | <,0001 | <,0001 | 0,1030 | <,0001 | 0,0440 | <,0001 | 0,0059 | 0,0276 | 0,7539 | <,0001 | <,0001 | <,0001 | 0,0133 | <,0001 | 0,0011 | <,0001 |
| Aβ5-40 | 0,0498 | 0,0264 | 0,6700 | 0,0440 | <,0001 | 0,0063 | 0,1241 | 0,1372 | 0,0348 | 0,3412 | 0,1300 | 0,0211 | 0,2429 | 0,0927 | 0,0430 | 0,0500 |
| Aβ4-40 | <,0001 | <,0001 | <,0001 | <,0001 | 0,0063 | <,0001 | 0,4494 | 0,0663 | 0,2410 | 0,0087 | 0,0011 | <,0001 | 0,4117 | 0,0200 | 0,0363 | 0,0213 |
| Aβ3pE-40 | 0,0011 | 0,0004 | 0,0181 | 0,0059 | 0,1241 | 0,4494 | <,0001 | <,0001 | 0,7857 | <,0001 | 0,0001 | <,0001 | <,0001 | <,0001 | <,0001 | <,0001 |
| Aβ3-40 | 0,0021 | 0,0041 | 0,3789 | 0,0276 | 0,1372 | 0,0663 | <,0001 | <,0001 | 0,5167 | 0,0004 | <,0001 | 0,0021 | <,0001 | <,0001 | <,0001 | <,0001 |
| Aβ2-40 | 0,8735 | 0,8473 | 0,4061 | 0,7539 | 0,0348 | 0,2410 | 0,7857 | 0,5167 | <,0001 | 0,2032 | 0,3065 | 0,7050 | 0,7503 | 0,4975 | 0,6765 | 0,4492 |
| Aβ1-40 | <,0001 | <,0001 | 0,9564 | <,0001 | 0,3412 | 0,0087 | <,0001 | 0,0004 | 0,2032 | <,0001 | <,0001 | <,0001 | <,0001 | <,0001 | <,0001 | <,0001 |
| Aβ11pE-42 | <,0001 | <,0001 | 0,6111 | <,0001 | 0,1300 | 0,0011 | 0,0001 | <,0001 | 0,3065 | <,0001 | <,0001 | <,0001 | <,0001 | <,0001 | <,0001 | <,0001 |
| Aβ11-42 | <,0001 | <,0001 | 0,4412 | <,0001 | 0,0211 | <,0001 | <,0001 | 0,0021 | 0,7050 | <,0001 | <,0001 | <,0001 | 0,0003 | <,0001 | <,0001 | <,0001 |
| Aβ5-42 | 0,0025 | 0,0025 | 0,0476 | 0,0133 | 0,2429 | 0,4117 | <,0001 | <,0001 | 0,7503 | <,0001 | <,0001 | 0,0003 | <,0001 | <,0001 | <,0001 | <,0001 |
| Aβ4-42 | <,0001 | <,0001 | 0,5605 | <,0001 | 0,0927 | 0,0200 | <,0001 | <,0001 | 0,4975 | <,0001 | <,0001 | <,0001 | <,0001 | <,0001 | <,0001 | <,0001 |
| Aβ3pE-42 | 0,0002 | 0,0003 | 0,3299 | 0,0011 | 0,0430 | 0,0363 | <,0001 | <,0001 | 0,6765 | <,0001 | <,0001 | <,0001 | <,0001 | <,0001 | <,0001 | <,0001 |
| Aβ1-42 | <,0001 | <,0001 | 0,4552 | <,0001 | 0,0500 | 0,0213 | <,0001 | <,0001 | 0,4492 | <,0001 | <,0001 | <,0001 | <,0001 | <,0001 | <,0001 | <,0001 |

### Scatterplot Matrix

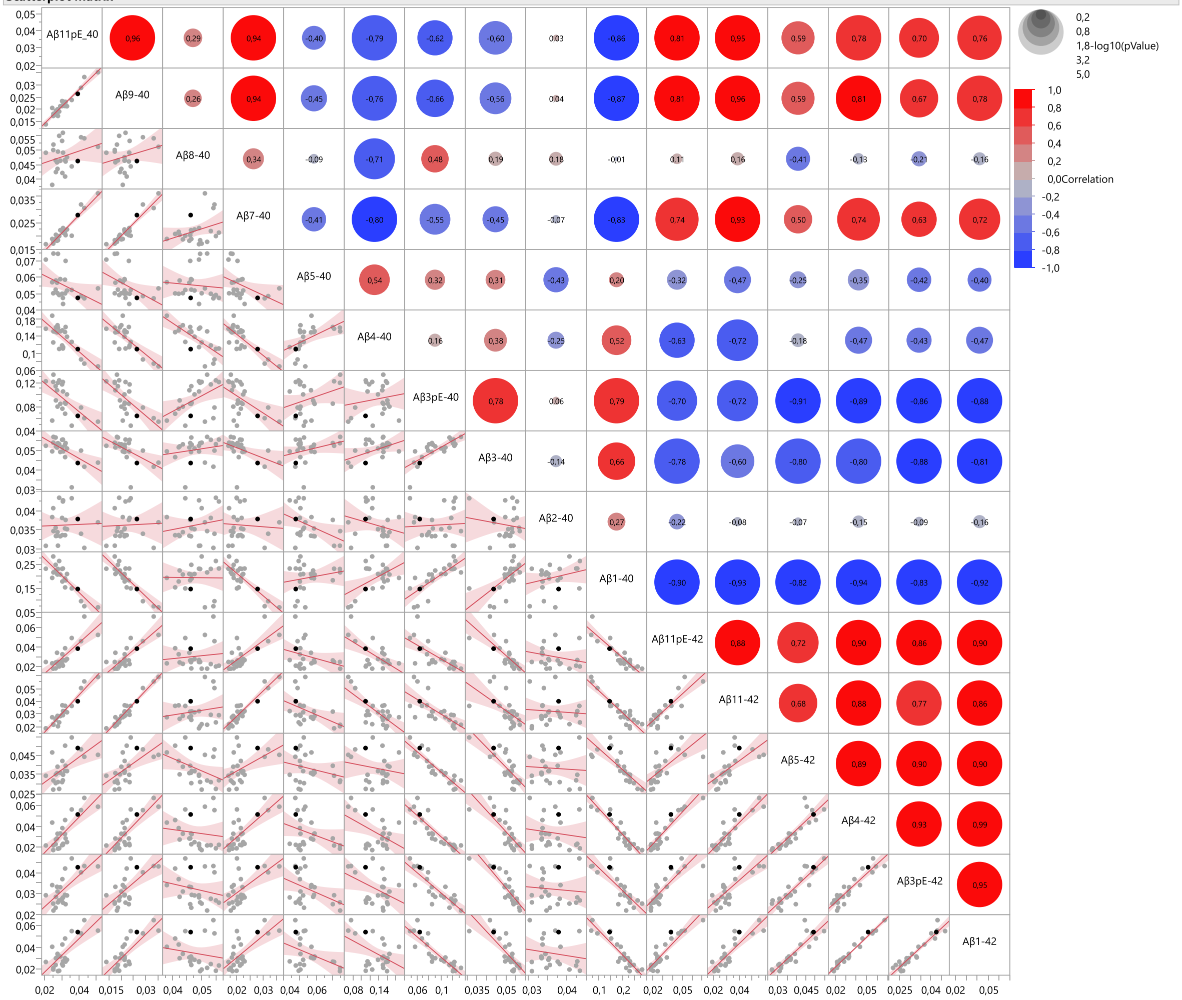


**Figure S8.**

Correlation analysis of amyloid peptides in sAD-Coarse Grained Plaques. Correlation matrix of all amyloid peptides indicating both correlation plots, significance (p value) and correlation $R^2$.

## Multivariate

### Correlations

| | Aβ11pE_40 | Aβ9-40 | Aβ8-40 | Aβ7-40 | Aβ5-40 | Aβ4-40 | Aβ3pE-40 | Aβ3-40 | Aβ2-40 | Aβ1-40 | Aβ11pE-42 | Aβ11-42 | Aβ5-42 | Aβ4-42 | Aβ3pE-42 | Aβ1-42 |
|---|---|---|---|---|---|---|---|---|---|---|---|---|---|---|---|---|
| Aβ11pE_40 | 1,0000 | 0,8476 | 0,2548 | 0,9054 | 0,7578 | 0,0458 | 0,0298 | 0,6744 | -0,7056 | -0,8947 | 0,5277 | 0,6436 | 0,8295 | 0,7449 | 0,5900 | 0,5758 |
| Aβ9-40 | 0,8476 | 1,0000 | 0,3217 | 0,9002 | 0,6593 | 0,0543 | 0,0083 | 0,4754 | -0,6247 | -0,9391 | 0,7362 | 0,8035 | 0,8028 | 0,8176 | 0,7533 | 0,7377 |
| Aβ8-40 | 0,2548 | 0,3217 | 1,0000 | 0,4231 | 0,3869 | 0,4132 | 0,3322 | 0,0593 | 0,2250 | -0,2539 | -0,1810 | -0,1230 | 0,0705 | -0,1011 | -0,0436 | -0,2561 |
| Aβ7-40 | 0,9054 | 0,9002 | 0,4231 | 1,0000 | 0,7156 | 0,1558 | 0,0783 | 0,5214 | -0,5453 | -0,9014 | 0,5872 | 0,6440 | 0,7882 | 0,7113 | 0,6212 | 0,5217 |
| Aβ5-40 | 0,7578 | 0,6593 | 0,3869 | 0,7156 | 1,0000 | 0,5056 | 0,3337 | 0,6935 | -0,6370 | -0,8052 | 0,3060 | 0,4019 | 0,7399 | 0,5416 | 0,3163 | 0,3743 |
| Aβ4-40 | 0,0458 | 0,0543 | 0,4132 | 0,1558 | 0,5056 | 1,0000 | 0,1626 | -0,0092 | -0,0156 | -0,1982 | -0,1262 | -0,1824 | 0,2571 | -0,0695 | -0,0489 | -0,1418 |
| Aβ3pE-40 | 0,0298 | 0,0083 | 0,3322 | 0,0783 | 0,3337 | 0,1626 | 1,0000 | 0,5066 | -0,1770 | -0,0102 | -0,3747 | -0,3351 | -0,2600 | -0,3189 | -0,4565 | -0,3719 |
| Aβ3-40 | 0,6744 | 0,4754 | 0,0593 | 0,5214 | 0,6935 | -0,0092 | 0,5066 | 1,0000 | -0,8267 | -0,5658 | 0,1249 | 0,2569 | 0,4260 | 0,3890 | 0,0222 | 0,2295 |
| Aβ2-40 | -0,7056 | -0,6247 | 0,2250 | -0,5453 | -0,6370 | -0,0156 | -0,1770 | -0,8267 | 1,0000 | 0,6956 | -0,4618 | -0,5418 | -0,6605 | -0,6428 | -0,3846 | -0,5912 |
| Aβ1-40 | -0,8947 | -0,9391 | -0,2539 | -0,9014 | -0,8052 | -0,1982 | -0,0102 | -0,5658 | 0,6956 | 1,0000 | -0,7567 | -0,8210 | -0,9140 | -0,8849 | -0,7499 | -0,7667 |
| Aβ11pE-42 | 0,5277 | 0,7362 | -0,1810 | 0,5872 | 0,3060 | -0,1262 | -0,3747 | 0,1249 | -0,4618 | -0,7567 | 1,0000 | 0,9743 | 0,7358 | 0,9260 | 0,9106 | 0,9488 |
| Aβ11-42 | 0,6436 | 0,8035 | -0,1230 | 0,6440 | 0,4019 | -0,1824 | -0,3351 | 0,2569 | -0,5418 | -0,8210 | 0,9743 | 1,0000 | 0,7821 | 0,9602 | 0,9013 | 0,9626 |
| Aβ5-42 | 0,8295 | 0,8028 | 0,0705 | 0,7882 | 0,7399 | 0,2571 | -0,2600 | 0,4260 | -0,6605 | -0,9140 | 0,7358 | 0,7821 | 1,0000 | 0,8791 | 0,8008 | 0,7601 |
| Aβ4-42 | 0,7449 | 0,8176 | -0,1011 | 0,7113 | 0,5416 | -0,0695 | -0,3189 | 0,3890 | -0,6428 | -0,8849 | 0,9260 | 0,9602 | 0,8791 | 1,0000 | 0,8700 | 0,9382 |
| Aβ3pE-42 | 0,5900 | 0,7533 | -0,0436 | 0,6212 | 0,3163 | -0,0489 | -0,4565 | 0,0222 | -0,3846 | -0,7499 | 0,9106 | 0,9013 | 0,8008 | 0,8700 | 1,0000 | 0,8616 |
| Aβ1-42 | 0,5758 | 0,7377 | -0,2561 | 0,5217 | 0,3743 | -0,1418 | -0,3719 | 0,2295 | -0,5912 | -0,7667 | 0,9488 | 0,9626 | 0,7601 | 0,9382 | 0,8616 | 1,0000 |

The correlations are estimated by Row-wise method.

### Correlation Probability

| | Aβ11pE_40 | Aβ9-40 | Aβ8-40 | Aβ7-40 | Aβ5-40 | Aβ4-40 | Aβ3pE-40 | Aβ3-40 | Aβ2-40 | Aβ1-40 | Aβ11pE-42 | Aβ11-42 | Aβ5-42 | Aβ4-42 | Aβ3pE-42 | Aβ1-42 |
|---|---|---|---|---|---|---|---|---|---|---|---|---|---|---|---|---|
| Aβ11pE_40 | <,0001 | <,0001 | 0,2783 | <,0001 | 0,0001 | 0,8479 | 0,9008 | 0,0011 | 0,0005 | <,0001 | 0,0168 | 0,0022 | <,0001 | 0,0002 | 0,0062 | 0,0079 |
| Aβ9-40 | <,0001 | <,0001 | 0,1666 | <,0001 | 0,0016 | 0,8203 | 0,9724 | 0,0341 | 0,0032 | <,0001 | 0,0002 | <,0001 | <,0001 | <,0001 | 0,0001 | 0,0002 |
| Aβ8-40 | 0,2783 | 0,1666 | <,0001 | 0,0631 | 0,0919 | 0,0702 | 0,1524 | 0,8038 | 0,3401 | 0,2802 | 0,4451 | 0,6054 | 0,7676 | 0,6713 | 0,8553 | 0,2758 |
| Aβ7-40 | <,0001 | <,0001 | 0,0631 | <,0001 | 0,0004 | 0,5119 | 0,7429 | 0,0184 | 0,0129 | <,0001 | 0,0065 | 0,0022 | <,0001 | 0,0004 | 0,0035 | 0,0183 |
| Aβ5-40 | 0,0001 | 0,0016 | 0,0919 | 0,0004 | <,0001 | 0,0230 | 0,1504 | 0,0007 | 0,0025 | <,0001 | 0,1895 | 0,0790 | 0,0002 | 0,0137 | 0,1742 | 0,1039 |
| Aβ4-40 | 0,8479 | 0,8203 | 0,0702 | 0,5119 | 0,0230 | <,0001 | 0,4935 | 0,9694 | 0,9478 | 0,4021 | 0,5960 | 0,4416 | 0,2739 | 0,7709 | 0,8379 | 0,5508 |
| Aβ3pE-40 | 0,9008 | 0,9724 | 0,1524 | 0,7429 | 0,1504 | 0,4935 | <,0001 | 0,0226 | 0,4553 | 0,9661 | 0,1035 | 0,1487 | 0,2684 | 0,1706 | 0,0430 | 0,1064 |
| Aβ3-40 | 0,0011 | 0,0341 | 0,8038 | 0,0184 | 0,0007 | 0,9694 | 0,0226 | <,0001 | <,0001 | 0,0093 | 0,5999 | 0,2742 | 0,0611 | 0,0901 | 0,9260 | 0,3304 |
| Aβ2-40 | 0,0005 | 0,0032 | 0,3401 | 0,0129 | 0,0025 | 0,9478 | 0,4553 | <,0001 | <,0001 | 0,0007 | 0,0404 | 0,0136 | 0,0015 | 0,0022 | 0,0941 | 0,0060 |
| Aβ1-40 | <,0001 | <,0001 | 0,2802 | <,0001 | <,0001 | 0,4021 | 0,9661 | 0,0093 | 0,0007 | <,0001 | 0,0001 | <,0001 | <,0001 | <,0001 | 0,0001 | <,0001 |
| Aβ11pE-42 | 0,0168 | 0,0002 | 0,4451 | 0,0065 | 0,1895 | 0,5960 | 0,1035 | 0,5999 | 0,0404 | 0,0001 | <,0001 | <,0001 | 0,0002 | <,0001 | <,0001 | <,0001 |
| Aβ11-42 | 0,0022 | <,0001 | 0,6054 | 0,0022 | 0,0790 | 0,4416 | 0,1487 | 0,2742 | 0,0136 | <,0001 | <,0001 | <,0001 | <,0001 | <,0001 | <,0001 | <,0001 |
| Aβ5-42 | <,0001 | <,0001 | 0,7676 | <,0001 | 0,0002 | 0,2739 | 0,2684 | 0,0611 | 0,0015 | <,0001 | 0,0002 | <,0001 | <,0001 | <,0001 | <,0001 | 0,0001 |
| Aβ4-42 | 0,0002 | <,0001 | 0,6713 | 0,0004 | 0,0137 | 0,7709 | 0,1706 | 0,0901 | 0,0022 | <,0001 | <,0001 | <,0001 | <,0001 | <,0001 | <,0001 | <,0001 |
| Aβ3pE-42 | 0,0062 | 0,0001 | 0,8553 | 0,0035 | 0,1742 | 0,8379 | 0,0430 | 0,9260 | 0,0941 | 0,0001 | <,0001 | <,0001 | <,0001 | <,0001 | <,0001 | <,0001 |
| Aβ1-42 | 0,0079 | 0,0002 | 0,2758 | 0,0183 | 0,1039 | 0,5508 | 0,1064 | 0,3304 | 0,0060 | <,0001 | <,0001 | <,0001 | 0,0001 | <,0001 | <,0001 | <,0001 |

### Scatterplot Matrix

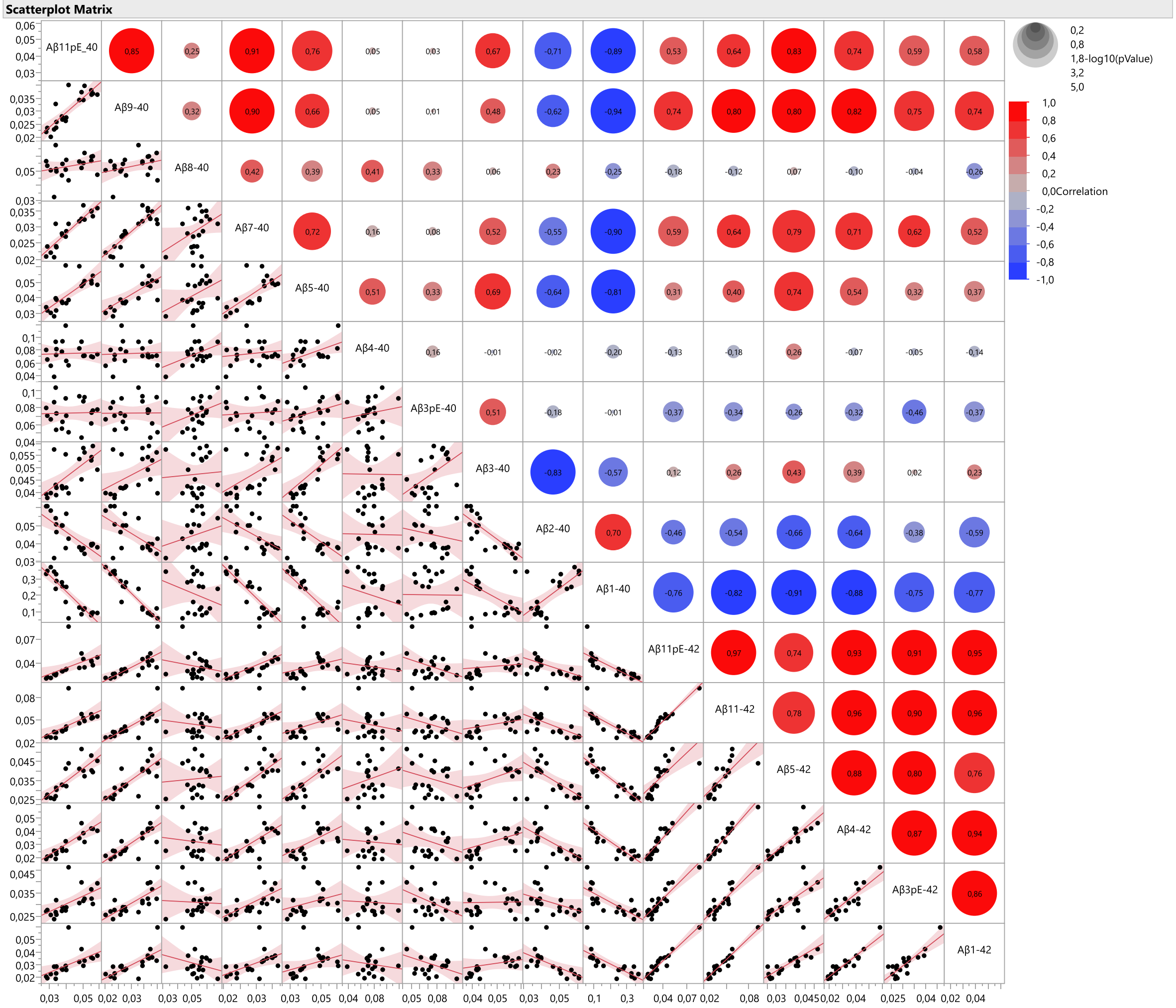


**Figure S9.**

Correlation analysis of amyloid peptides in fAD-Coarse Grained Plaques. Correlation matrix of all amyloid peptides indicating both correlation plots, significance (p value) and correlation $R^2$.

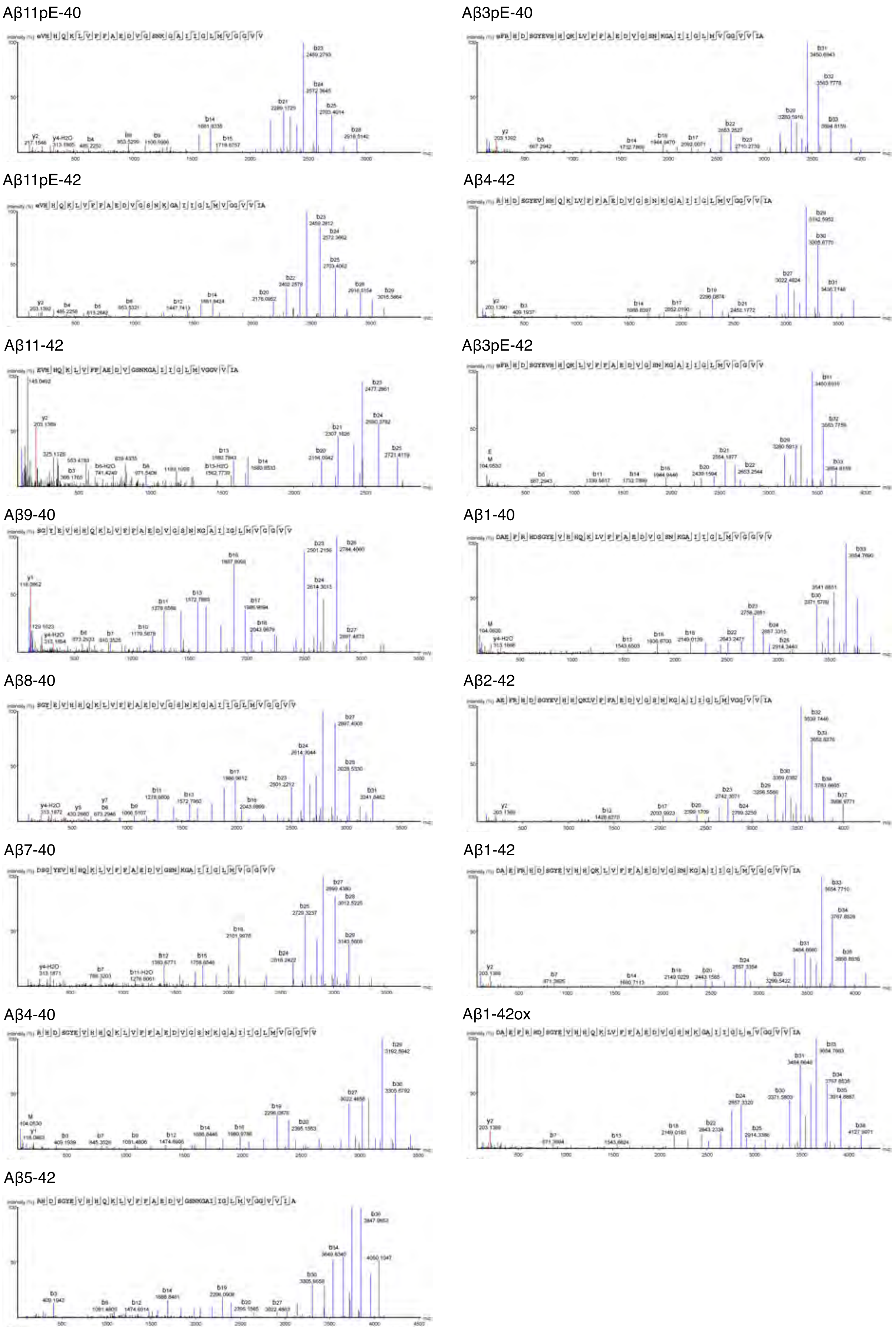


**Figure S10. LC-MS/MS validation of peptide masses detected *in situ*.**

Orbitrap MS/MS for different Aβ peptides. Peptide fragment and sequence annotation performed in Peaks illustrate the sequence coverage and successful identification.

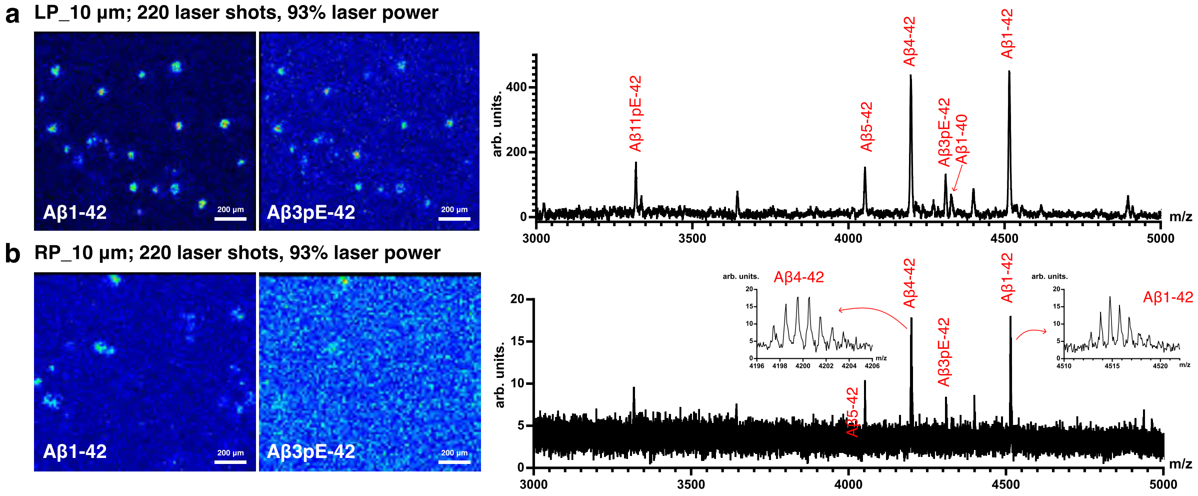


**Figure S11. Effect of MALDI TOF detection mode on sensitivity.**

Single ion images and single plaque spectra for MSI data acquired **a** in linear mode (LP) **b** and reflective mode (RP) acquired with the same spatial resolution and laser energy. Scalebar: 200µm.

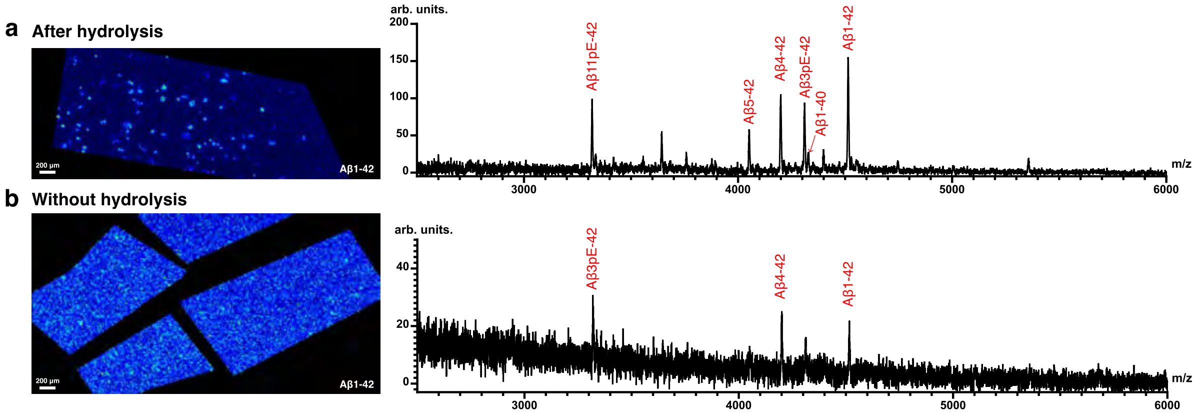


**Figure S12. Effect of sample hydrolysis on amyloid plaque pattern.**

**a** MALDI imaging results for a tissue section prepared using formic acid hydrolysis. Corresponding single ion image and plaque ROI spectra show Aβ species including Aβ11pE-42, Aβ5-42, Aβ4-42, Aβ3pE-42, Aβ1-40, and Aβ1-42. **b** MALDI imaging results for a tissue section prepared without formic acid hydrolysis. Detected Aβ species in this condition include Aβ3pE-42, Aβ4-42 and Aβ1-42.

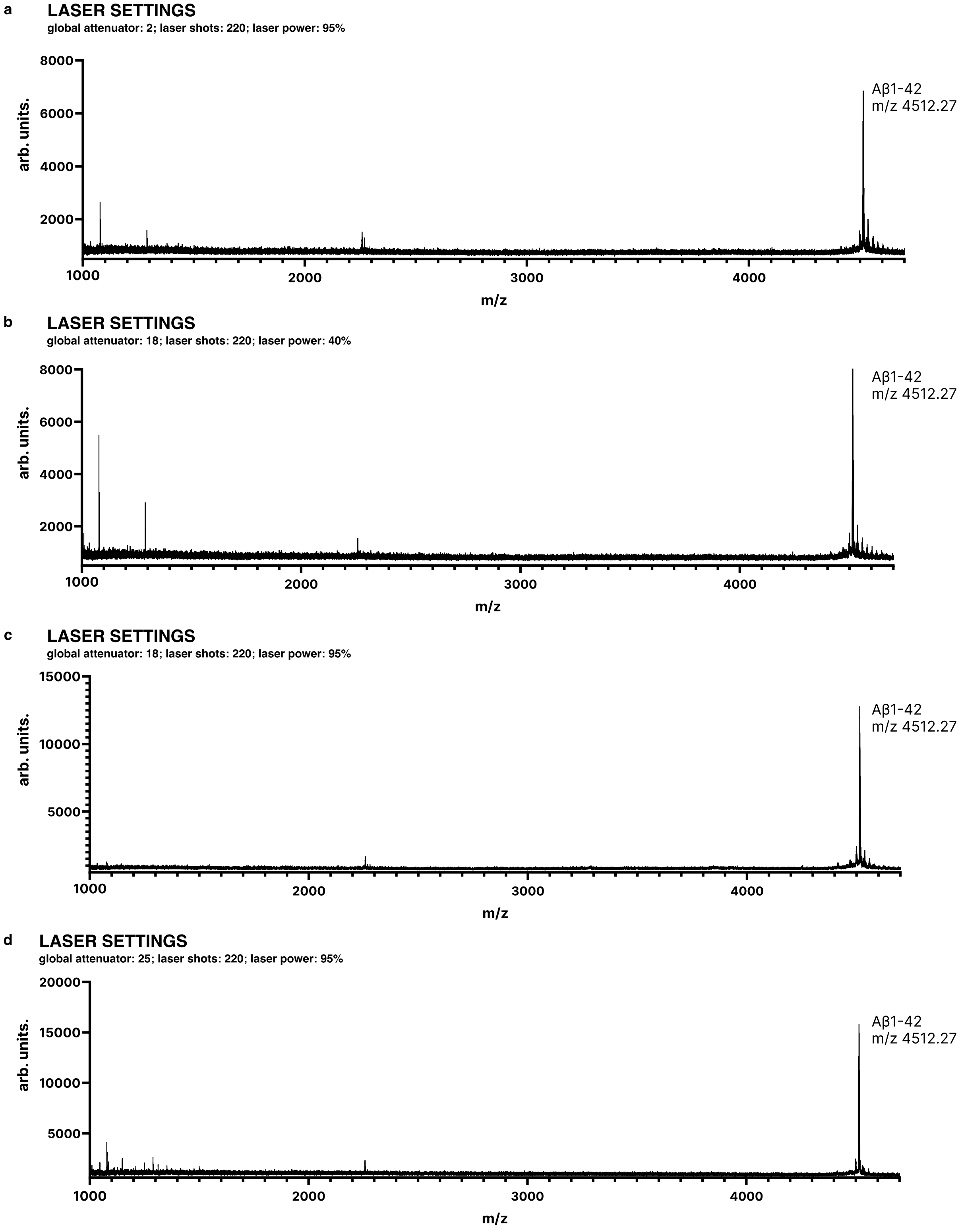


**Figure S13. Effect of laser fluence on amyloid peptide in source fragmentation.**

The effect of laser fluence was tested by spotting a synthetic Aβ1-42 peptide standard onto the tissue sample. The global attenuator offset and relative laser power was incrementally increased from the acquisition settings **a** using otherwise same criteria for MSI data acquisition (220shots, laser small). No Aβ42 related fragments was observed for **a-c**, while some minor fragments were detected for the maximum laser power setting **d**.

## Multivariate

### Correlations

| | Aβ9-40 | Aβ4-40 | Aβ3pE-40 | Aβ1-40 | Aβ11pE-42 | Aβ5-42 | Aβ4-42 | Aβ3pE-42 | Aβ2-42 | Aβ1-42 | Aβ1-42ox |
|---|---|---|---|---|---|---|---|---|---|---|---|
| Aβ9-40 | 1,0000 | 0,4551 | 0,0156 | 0,0266 | 0,0733 | -0,2650 | -0,1016 | -0,0897 | 0,0612 | 0,0433 | -0,1377 |
| Aβ4-40 | 0,4551 | 1,0000 | 0,0798 | -0,1682 | -0,2693 | -0,0844 | -0,0615 | -0,3898 | -0,2017 | -0,2093 | -0,3037 |
| Aβ3pE-40 | 0,0156 | 0,0798 | 1,0000 | -0,3712 | -0,0946 | -0,3260 | -0,5046 | -0,1014 | -0,0529 | -0,4085 | -0,4098 |
| Aβ1-40 | 0,0266 | -0,1682 | -0,3712 | 1,0000 | 0,7078 | 0,4375 | 0,5081 | 0,8085 | 0,8820 | 0,9291 | 0,6656 |
| Aβ11pE-42 | 0,0733 | -0,2693 | -0,0946 | 0,7078 | 1,0000 | 0,1847 | 0,2094 | 0,7238 | 0,6599 | 0,6668 | 0,4486 |
| Aβ5-42 | -0,2650 | -0,0844 | -0,3260 | 0,4375 | 0,1847 | 1,0000 | 0,7822 | 0,2463 | 0,1771 | 0,4180 | 0,2599 |
| Aβ4-42 | -0,1016 | -0,0615 | -0,5046 | 0,5081 | 0,2094 | 0,7822 | 1,0000 | 0,1105 | 0,3021 | 0,5872 | 0,3508 |
| Aβ3pE-42 | -0,0897 | -0,3898 | -0,1014 | 0,8085 | 0,7238 | 0,2463 | 0,1105 | 1,0000 | 0,7582 | 0,7133 | 0,6517 |
| Aβ2-42 | 0,0612 | -0,2017 | -0,0529 | 0,8820 | 0,6599 | 0,1771 | 0,3021 | 0,7582 | 1,0000 | 0,9017 | 0,6453 |
| Aβ1-42 | 0,0433 | -0,2093 | -0,4085 | 0,9291 | 0,6668 | 0,4180 | 0,5872 | 0,7133 | 0,9017 | 1,0000 | 0,7498 |
| Aβ1-42ox | -0,1377 | -0,3037 | -0,4098 | 0,6656 | 0,4486 | 0,2599 | 0,3508 | 0,6517 | 0,6453 | 0,7498 | 1,0000 |

There are 10 missing values.The correlations are estimated by Pairwise method.

### Correlation Probability

| | Aβ9-40 | Aβ4-40 | Aβ3pE-40 | Aβ1-40 | Aβ11pE-42 | Aβ5-42 | Aβ4-42 | Aβ3pE-42 | Aβ2-42 | Aβ1-42 | Aβ1-42ox |
|---|---|---|---|---|---|---|---|---|---|---|---|
| Aβ9-40 | <,0001 | 0,0089 | 0,9315 | 0,8832 | 0,6850 | 0,1361 | 0,5739 | 0,6195 | 0,7393 | 0,8079 | 0,4448 |
| Aβ4-40 | 0,0089 | <,0001 | 0,6536 | 0,3575 | 0,1360 | 0,6461 | 0,7381 | 0,0274 | 0,2603 | 0,2425 | 0,0808 |
| Aβ3pE-40 | 0,9315 | 0,6536 | <,0001 | 0,0398 | 0,6128 | 0,0641 | 0,0027 | 0,5874 | 0,7665 | 0,0203 | 0,0145 |
| Aβ1-40 | 0,8832 | 0,3575 | 0,0398 | <,0001 | <,0001 | 0,0109 | 0,0035 | <,0001 | <,0001 | <,0001 | <,0001 |
| Aβ11pE-42 | 0,6850 | 0,1360 | 0,6128 | <,0001 | <,0001 | 0,3036 | 0,2581 | <,0001 | <,0001 | <,0001 | 0,0114 |
| Aβ5-42 | 0,1361 | 0,6461 | 0,0641 | 0,0109 | 0,3036 | <,0001 | <,0001 | 0,1816 | 0,3323 | 0,0173 | 0,1442 |
| Aβ4-42 | 0,5739 | 0,7381 | 0,0027 | 0,0035 | 0,2581 | <,0001 | <,0001 | 0,5403 | 0,0825 | 0,0004 | 0,0453 |
| Aβ3pE-42 | 0,6195 | 0,0274 | 0,5874 | <,0001 | <,0001 | 0,1816 | 0,5403 | <,0001 | <,0001 | <,0001 | <,0001 |
| Aβ2-42 | 0,7393 | 0,2603 | 0,7665 | <,0001 | <,0001 | 0,3323 | 0,0825 | <,0001 | <,0001 | <,0001 | <,0001 |
| Aβ1-42 | 0,8079 | 0,2425 | 0,0203 | <,0001 | <,0001 | 0,0173 | 0,0004 | <,0001 | <,0001 | <,0001 | <,0001 |
| Aβ1-42ox | 0,4448 | 0,0808 | 0,0145 | <,0001 | 0,0114 | 0,1442 | 0,0453 | <,0001 | <,0001 | <,0001 | <,0001 |

### Scatterplot Matrix

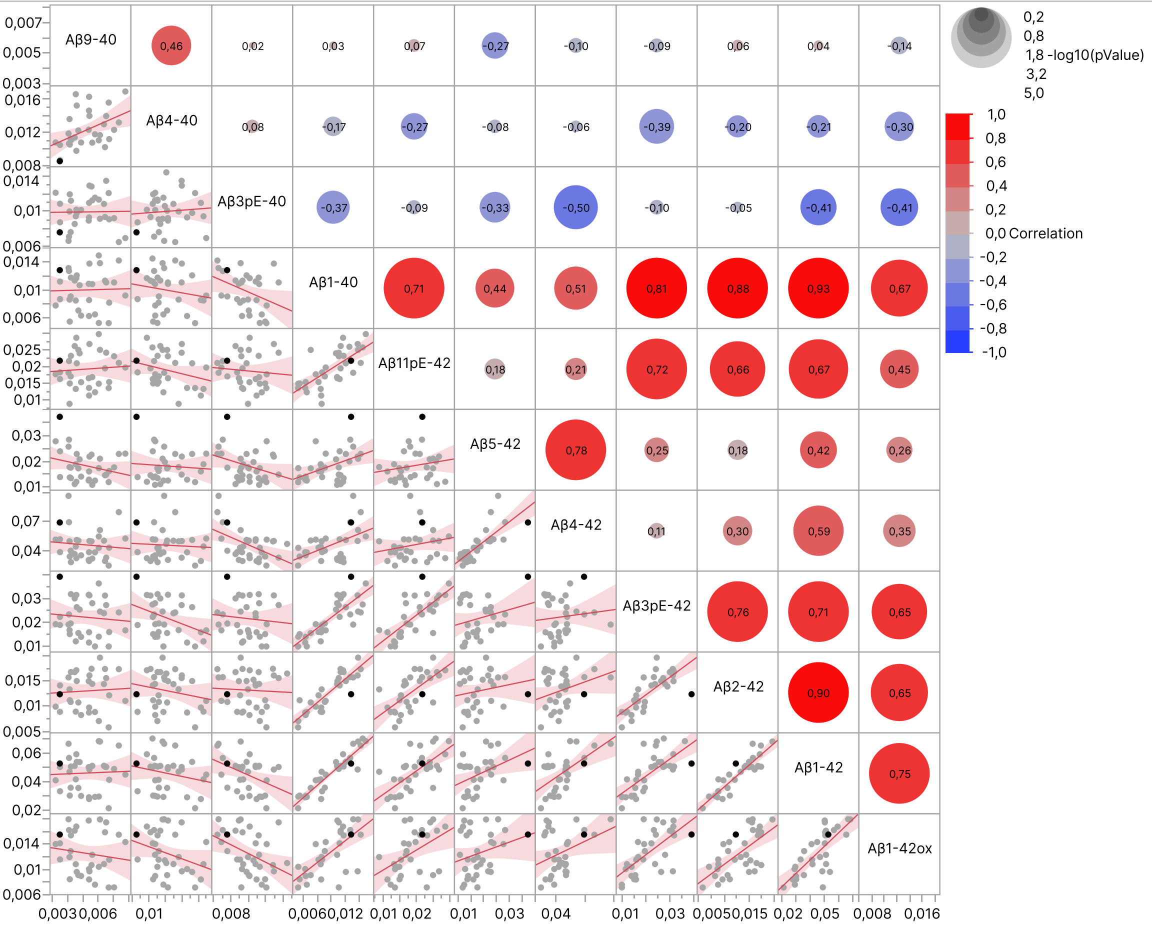


**Figure S14.**

Correlation analysis of amyloid peptides in CUAP-Cored Plaques. Correlation matrix of all amyloid peptides indicating both correlation plots, significance (p value) and correlation $R^2$.

## Multivariate

### Correlations

| | Aβ9-40 | Aβ4-40 | Aβ3pE-40 | Aβ1-40 | Aβ11pE-42 | Aβ5-42 | Aβ4-42 | Aβ3pE-42 | Aβ2-42 | Aβ1-42 | Aβ1-42ox |
|---|---|---|---|---|---|---|---|---|---|---|---|
| Aβ9-40 | 1,0000 | -0,1125 | -0,0285 | -0,3031 | 0,0416 | -0,1562 | -0,2297 | -0,2278 | -0,2016 | -0,2005 | -0,0800 |
| Aβ4-40 | -0,1125 | 1,0000 | 0,0081 | -0,0053 | 0,2369 | -0,2117 | -0,2411 | -0,2095 | -0,1313 | -0,0803 | -0,0380 |
| Aβ3pE-40 | -0,0285 | 0,0081 | 1,0000 | -0,4861 | -0,1945 | -0,4998 | -0,3131 | -0,6722 | -0,4967 | -0,4979 | -0,3723 |
| Aβ1-40 | -0,3031 | -0,0053 | -0,4861 | 1,0000 | 0,4041 | 0,4615 | 0,4929 | 0,5210 | 0,7144 | 0,7577 | 0,6201 |
| Aβ11pE-42 | 0,0416 | 0,2369 | -0,1945 | 0,4041 | 1,0000 | 0,1424 | -0,0882 | 0,2590 | 0,5484 | 0,5014 | -0,1097 |
| Aβ5-42 | -0,1562 | -0,2117 | -0,4998 | 0,4615 | 0,1424 | 1,0000 | 0,8784 | 0,6246 | 0,4858 | 0,5946 | 0,4339 |
| Aβ4-42 | -0,2297 | -0,2411 | -0,3131 | 0,4929 | -0,0882 | 0,8784 | 1,0000 | 0,4262 | 0,5481 | 0,6444 | 0,4182 |
| Aβ3pE-42 | -0,2278 | -0,2095 | -0,6722 | 0,5210 | 0,2590 | 0,6246 | 0,4262 | 1,0000 | 0,5678 | 0,6126 | 0,3004 |
| Aβ2-42 | -0,2016 | -0,1313 | -0,4967 | 0,7144 | 0,5484 | 0,4858 | 0,5481 | 0,5678 | 1,0000 | 0,8377 | 0,4657 |
| Aβ1-42 | -0,2005 | -0,0803 | -0,4979 | 0,7577 | 0,5014 | 0,5946 | 0,6444 | 0,6126 | 0,8377 | 1,0000 | 0,7176 |
| Aβ1-42ox | -0,0800 | -0,0380 | -0,3723 | 0,6201 | -0,1097 | 0,4339 | 0,4182 | 0,3004 | 0,4657 | 0,7176 | 1,0000 |

There are 6 missing values. The correlations are estimated by Pairwise method.

### Correlation Probability

| | Aβ9-40 | Aβ4-40 | Aβ3pE-40 | Aβ1-40 | Aβ11pE-42 | Aβ5-42 | Aβ4-42 | Aβ3pE-42 | Aβ2-42 | Aβ1-42 | Aβ1-42ox |
|---|---|---|---|---|---|---|---|---|---|---|---|
| Aβ9-40 | <,0001 | 0,5263 | 0,8770 | 0,0864 | 0,8183 | 0,3776 | 0,1985 | 0,1882 | 0,2529 | 0,2631 | 0,6528 |
| Aβ4-40 | 0,5263 | <,0001 | 0,9645 | 0,9762 | 0,1917 | 0,2220 | 0,1838 | 0,2344 | 0,4521 | 0,6516 | 0,8337 |
| Aβ3pE-40 | 0,8770 | 0,9645 | <,0001 | 0,0036 | 0,2702 | 0,0031 | 0,0714 | <,0001 | 0,0033 | 0,0027 | 0,0329 |
| Aβ1-40 | 0,0864 | 0,9762 | 0,0036 | <,0001 | 0,0197 | 0,0060 | 0,0036 | 0,0019 | <,0001 | <,0001 | <,0001 |
| Aβ11pE-42 | 0,8183 | 0,1917 | 0,2702 | 0,0197 | <,0001 | 0,4369 | 0,6144 | 0,1455 | 0,0012 | 0,0030 | 0,5368 |
| Aβ5-42 | 0,3776 | 0,2220 | 0,0031 | 0,0060 | 0,4369 | <,0001 | <,0001 | <,0001 | 0,0031 | 0,0002 | 0,0116 |
| Aβ4-42 | 0,1985 | 0,1838 | 0,0714 | 0,0036 | 0,6144 | <,0001 | <,0001 | 0,0134 | 0,0012 | <,0001 | 0,0138 |
| Aβ3pE-42 | 0,1882 | 0,2344 | <,0001 | 0,0019 | 0,1455 | <,0001 | 0,0134 | <,0001 | 0,0005 | 0,0002 | 0,0843 |
| Aβ2-42 | 0,2529 | 0,4521 | 0,0033 | <,0001 | 0,0012 | 0,0031 | 0,0012 | 0,0005 | <,0001 | <,0001 | 0,0063 |
| Aβ1-42 | 0,2631 | 0,6516 | 0,0027 | <,0001 | 0,0030 | 0,0002 | <,0001 | 0,0002 | <,0001 | <,0001 | <,0001 |
| Aβ1-42ox | 0,6528 | 0,8337 | 0,0329 | <,0001 | 0,5368 | 0,0116 | 0,0138 | 0,0843 | 0,0063 | <,0001 | <,0001 |

### Scatterplot Matrix

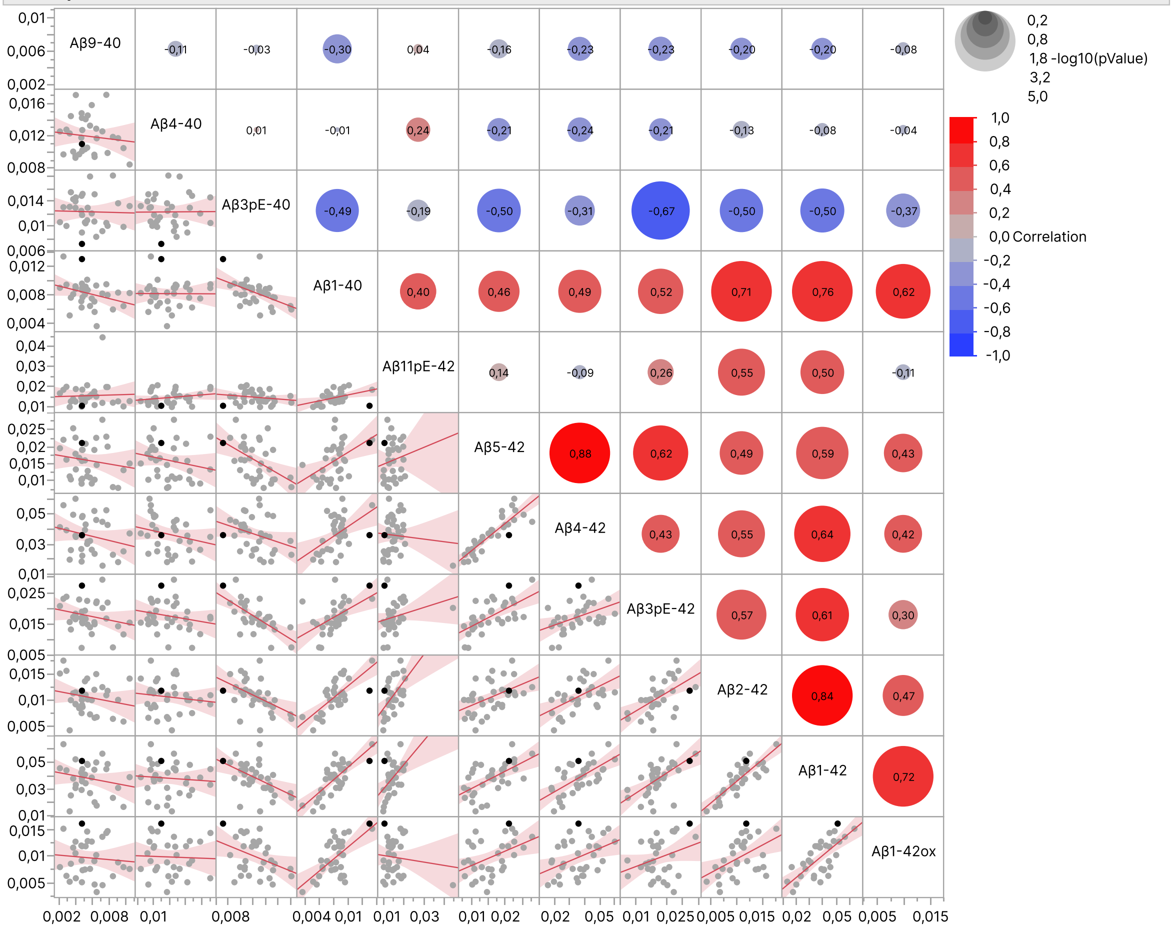


**Figure S15.**

Correlation analysis of amyloid peptides in CUAP-Diffuse Plaques. Correlation matrix of all amyloid peptides indicating both correlation plots, significance (p value) and correlation $R^2$.

**Multivariate**

**Correlations**

| | Aβ11pE-42 | Aβ11-42 | Aβ9-40 | Aβ4-40 | Aβ5-42 | Aβ3pE-40 | Aβ4-42 | Aβ4-42ox | Aβ3pE-42 | Aβ1-40 | Aβ2-42 | Aβ1-42 | Aβ1-42ox |
|---|---|---|---|---|---|---|---|---|---|---|---|---|---|
| Aβ11pE-42 | 1,0000 | 0,6621 | 0,0464 | 0,2553 | -0,1013 | -0,1145 | -0,5160 | -0,1102 | 0,5032 | -0,5490 | -0,0906 | -0,4979 | 0,0480 |
| Aβ11-42 | 0,6621 | 1,0000 | 0,4022 | 0,2963 | -0,1595 | -0,2740 | 0,1925 | 0,3156 | 0,7159 | -0,7344 | 0,4154 | -0,3916 | -0,2414 |
| Aβ9-40 | 0,0464 | 0,4022 | 1,0000 | 0,3643 | -0,0133 | -0,0089 | 0,6395 | 0,5154 | -0,1058 | -0,5962 | 0,1854 | -0,1143 | -0,1409 |
| Aβ4-40 | 0,2553 | 0,2963 | 0,3643 | 1,0000 | -0,0017 | -0,4835 | -0,0329 | -0,3124 | -0,0597 | -0,4075 | -0,2919 | -0,4781 | -0,4363 |
| Aβ5-42 | -0,1013 | -0,1595 | -0,0133 | -0,0017 | 1,0000 | -0,4886 | -0,1890 | -0,2126 | -0,1470 | 0,2921 | -0,3432 | -0,2180 | 0,0955 |
| Aβ3pE-40 | -0,1145 | -0,2740 | -0,0089 | -0,4835 | -0,4886 | 1,0000 | 0,0487 | 0,0885 | -0,2706 | 0,1160 | 0,2435 | 0,0755 | -0,1255 |
| Aβ4-42 | -0,5160 | 0,1925 | 0,6395 | -0,0329 | -0,1890 | 0,0487 | 1,0000 | 0,7216 | -0,0998 | -0,1894 | 0,6049 | 0,2497 | -0,2523 |
| Aβ4-42ox | -0,1102 | 0,3156 | 0,5154 | -0,3124 | -0,2126 | 0,0885 | 0,7216 | 1,0000 | 0,2004 | -0,4441 | 0,3986 | 0,4477 | 0,1476 |
| Aβ3pE-42 | 0,5032 | 0,7159 | -0,1058 | -0,0597 | -0,1470 | -0,2706 | -0,0998 | 0,2004 | 1,0000 | -0,5873 | 0,0987 | 0,1130 | 0,1788 |
| Aβ1-40 | -0,5490 | -0,7344 | -0,5962 | -0,4075 | 0,2921 | 0,1160 | -0,1894 | -0,4441 | -0,5873 | 1,0000 | 0,0693 | 0,0118 | 0,0091 |
| Aβ2-42 | -0,0906 | 0,4154 | 0,1854 | -0,2919 | -0,3432 | 0,2435 | 0,6049 | 0,3986 | 0,0987 | 0,0693 | 1,0000 | -0,1823 | -0,3816 |
| Aβ1-42 | -0,4979 | -0,3916 | -0,1143 | -0,4781 | -0,2180 | 0,0755 | 0,2497 | 0,4477 | 0,1130 | 0,0118 | -0,1823 | 1,0000 | 0,6695 |
| Aβ1-42ox | 0,0480 | -0,2414 | -0,1409 | -0,4363 | 0,0955 | -0,1255 | -0,2523 | 0,1476 | 0,1788 | 0,0091 | -0,3816 | 0,6695 | 1,0000 |

The correlations are estimated by Row-wise method.

**Correlation Probability**

| | Aβ11pE-42 | Aβ11-42 | Aβ9-40 | Aβ4-40 | Aβ5-42 | Aβ3pE-40 | Aβ4-42 | Aβ4-42ox | Aβ3pE-42 | Aβ1-40 | Aβ2-42 | Aβ1-42 | Aβ1-42ox |
|---|---|---|---|---|---|---|---|---|---|---|---|---|---|
| Aβ11pE-42 | <,0001 | 0,0520 | 0,9056 | 0,5073 | 0,7953 | 0,7693 | 0,1550 | 0,7777 | 0,1673 | 0,1258 | 0,8167 | 0,1726 | 0,9024 |
| Aβ11-42 | 0,0520 | <,0001 | 0,2832 | 0,4387 | 0,6818 | 0,4756 | 0,6197 | 0,4080 | 0,0301 | 0,0243 | 0,2661 | 0,2972 | 0,5314 |
| Aβ9-40 | 0,9056 | 0,2832 | <,0001 | 0,3351 | 0,9729 | 0,9819 | 0,0637 | 0,1556 | 0,7865 | 0,0902 | 0,6329 | 0,7697 | 0,7176 |
| Aβ4-40 | 0,5073 | 0,4387 | 0,3351 | <,0001 | 0,9966 | 0,1873 | 0,9331 | 0,4131 | 0,8787 | 0,2763 | 0,4460 | 0,1930 | 0,2404 |
| Aβ5-42 | 0,7953 | 0,6818 | 0,9729 | 0,9966 | <,0001 | 0,1820 | 0,6263 | 0,5828 | 0,7059 | 0,4457 | 0,3659 | 0,5732 | 0,8070 |
| Aβ3pE-40 | 0,7693 | 0,4756 | 0,9819 | 0,1873 | 0,1820 | <,0001 | 0,9010 | 0,8209 | 0,4812 | 0,7662 | 0,5278 | 0,8470 | 0,7477 |
| Aβ4-42 | 0,1550 | 0,6197 | 0,0637 | 0,9331 | 0,6263 | 0,9010 | <,0001 | 0,0282 | 0,7984 | 0,6255 | 0,0844 | 0,5169 | 0,5124 |
| Aβ4-42ox | 0,7777 | 0,4080 | 0,1556 | 0,4131 | 0,5828 | 0,8209 | 0,0282 | <,0001 | 0,6052 | 0,2311 | 0,2879 | 0,2269 | 0,7048 |
| Aβ3pE-42 | 0,1673 | 0,0301 | 0,7865 | 0,8787 | 0,7059 | 0,4812 | 0,7984 | 0,6052 | <,0001 | 0,0964 | 0,8005 | 0,7722 | 0,6453 |
| Aβ1-40 | 0,1258 | 0,0243 | 0,0902 | 0,2763 | 0,4457 | 0,7662 | 0,6255 | 0,2311 | 0,0964 | <,0001 | 0,8595 | 0,9759 | 0,9815 |
| Aβ2-42 | 0,8167 | 0,2661 | 0,6329 | 0,4460 | 0,3659 | 0,5278 | 0,0844 | 0,2879 | 0,8005 | 0,8595 | <,0001 | 0,6387 | 0,3108 |
| Aβ1-42 | 0,1726 | 0,2972 | 0,7697 | 0,1930 | 0,5732 | 0,8470 | 0,5169 | 0,2269 | 0,7722 | 0,9759 | 0,6387 | <,0001 | 0,0485 |
| Aβ1-42ox | 0,9024 | 0,5314 | 0,7176 | 0,2404 | 0,8070 | 0,7477 | 0,5124 | 0,7048 | 0,6453 | 0,9815 | 0,3108 | 0,0485 | <,0001 |

**Scatterplot Matrix**

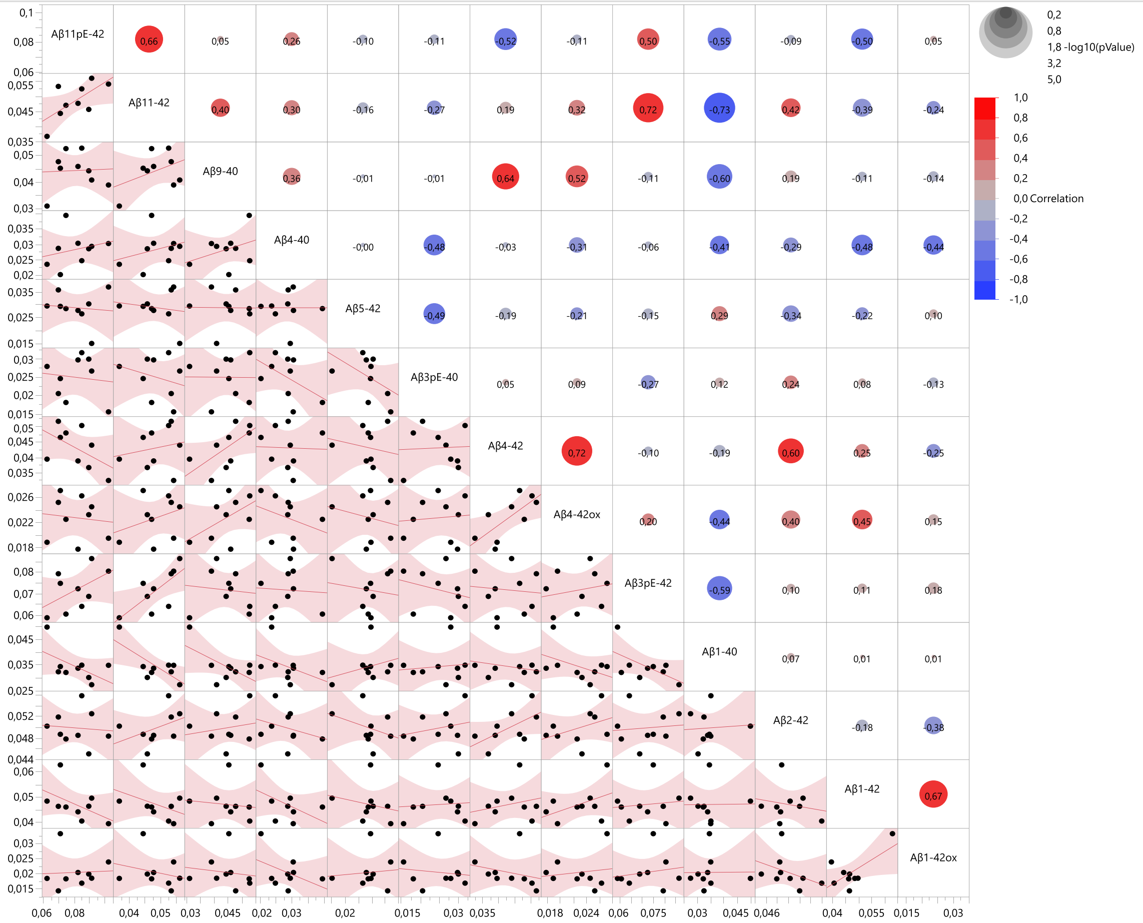


## Figure S16.

Correlation analysis of amyloid peptides in Cotton Wool Plaques. Correlation matrix of all amyloid peptides indicating both correlation plots, significance (p value) and correlation $R^2$.

## Multivariate

### Correlations

| | Aβ11pE_40 | Aβ9-40 | Aβ8-40 | Aβ7-40 | Aβ5-40 | Aβ4-40 | Aβ3pE-40 | Aβ3-40 | Aβ2-40 | Aβ1-40 | Aβ11pE-42 | Aβ11-42 | Aβ5-42 | Aβ4-42 | Aβ3pE-42 | Aβ1-42 |
|---|---|---|---|---|---|---|---|---|---|---|---|---|---|---|---|---|
| Aβ11pE_40 | 1,0000 | 0,8803 | 0,6059 | 0,8137 | 0,3495 | -0,4063 | 0,1208 | 0,1337 | -0,4686 | -0,4517 | 0,2769 | 0,3102 | -0,2344 | -0,0189 | -0,4970 | 0,2946 |
| Aβ9-40 | 0,8803 | 1,0000 | 0,6406 | 0,8791 | 0,0915 | -0,4150 | -0,0423 | -0,1118 | -0,2977 | -0,3748 | 0,4415 | 0,4085 | -0,2932 | -0,2823 | -0,3493 | 0,1800 |
| Aβ8-40 | 0,6059 | 0,6406 | 1,0000 | 0,5140 | 0,5601 | -0,0635 | 0,2110 | 0,0170 | -0,5393 | -0,4761 | 0,1491 | 0,1030 | -0,1312 | -0,3120 | -0,0861 | -0,3927 |
| Aβ7-40 | 0,8137 | 0,8791 | 0,5140 | 1,0000 | 0,0473 | -0,4184 | -0,2058 | -0,2097 | -0,2656 | -0,3495 | 0,5314 | 0,4950 | -0,1953 | -0,2691 | -0,3921 | 0,3351 |
| Aβ5-40 | 0,3495 | 0,0915 | 0,5601 | 0,0473 | 1,0000 | 0,3222 | 0,3810 | 0,3725 | -0,8018 | -0,5505 | -0,0794 | 0,0392 | 0,1619 | 0,2325 | -0,3734 | -0,0823 |
| Aβ4-40 | -0,4063 | -0,4150 | -0,0635 | -0,4184 | 0,3222 | 1,0000 | -0,3585 | -0,0772 | -0,2994 | -0,3530 | 0,3062 | 0,2541 | 0,6572 | 0,0358 | 0,0835 | -0,3603 |
| Aβ3pE-40 | 0,1208 | -0,0423 | 0,2110 | -0,2058 | 0,3810 | -0,3585 | 1,0000 | 0,7939 | -0,0042 | -0,1559 | -0,6900 | -0,5078 | -0,1991 | 0,4338 | -0,0322 | -0,0193 |
| Aβ3-40 | 0,1337 | -0,1118 | 0,0170 | -0,2097 | 0,3725 | -0,0772 | 0,7939 | 1,0000 | -0,0645 | -0,4491 | -0,4989 | -0,1958 | 0,2761 | 0,7379 | -0,1522 | 0,1338 |
| Aβ2-40 | -0,4686 | -0,2977 | -0,5393 | -0,2656 | -0,8018 | -0,2994 | -0,0042 | -0,0645 | 1,0000 | 0,3891 | -0,1632 | -0,2439 | -0,0731 | -0,0839 | 0,4418 | 0,0306 |
| Aβ1-40 | -0,4517 | -0,3748 | -0,4761 | -0,3495 | -0,5505 | -0,3530 | -0,1559 | -0,4491 | 0,3891 | 1,0000 | -0,4266 | -0,5451 | -0,5988 | -0,3218 | 0,2410 | -0,0612 |
| Aβ11pE-42 | 0,2769 | 0,4415 | 0,1491 | 0,5314 | -0,0794 | 0,3062 | -0,6900 | -0,4989 | -0,1632 | -0,4266 | 1,0000 | 0,8502 | 0,3440 | -0,3491 | -0,2263 | 0,0796 |
| Aβ11-42 | 0,3102 | 0,4085 | 0,1030 | 0,4950 | 0,0392 | 0,2541 | -0,5078 | -0,1958 | -0,2439 | -0,5451 | 0,8502 | 1,0000 | 0,4805 | -0,0123 | -0,4641 | 0,2289 |
| Aβ5-42 | -0,2344 | -0,2932 | -0,1312 | -0,1953 | 0,1619 | 0,6572 | -0,1991 | 0,2761 | -0,0731 | -0,5988 | 0,3440 | 0,4805 | 1,0000 | 0,4101 | 0,0093 | -0,1359 |
| Aβ4-42 | -0,0189 | -0,2823 | -0,3120 | -0,2691 | 0,2325 | 0,0358 | 0,4338 | 0,7379 | -0,0839 | -0,3218 | -0,3491 | -0,0123 | 0,4101 | 1,0000 | -0,0894 | 0,4700 |
| Aβ3pE-42 | -0,4970 | -0,3493 | -0,0861 | -0,3921 | -0,3734 | 0,0835 | -0,0322 | -0,1522 | 0,4418 | 0,2410 | -0,2263 | -0,4641 | 0,0093 | -0,0894 | 1,0000 | -0,3311 |
| Aβ1-42 | 0,2946 | 0,1800 | -0,3927 | 0,3351 | -0,0823 | -0,3603 | -0,0193 | 0,1338 | 0,0306 | -0,0612 | 0,0796 | 0,2289 | -0,1359 | 0,4700 | -0,3311 | 1,0000 |

The correlations are estimated by Row-wise method.

### Correlation Probability

| | Aβ11pE_40 | Aβ9-40 | Aβ8-40 | Aβ7-40 | Aβ5-40 | Aβ4-40 | Aβ3pE-40 | Aβ3-40 | Aβ2-40 | Aβ1-40 | Aβ11pE-42 | Aβ11-42 | Aβ5-42 | Aβ4-42 | Aβ3pE-42 | Aβ1-42 |
|---|---|---|---|---|---|---|---|---|---|---|---|---|---|---|---|---|
| Aβ11pE_40 | <,0001 | <,0001 | 0,0028 | <,0001 | 0,1109 | 0,0606 | 0,5924 | 0,5530 | 0,0278 | 0,0348 | 0,2122 | 0,1600 | 0,2937 | 0,9334 | 0,0186 | 0,1831 |
| Aβ9-40 | <,0001 | <,0001 | 0,0013 | <,0001 | 0,6854 | 0,0548 | 0,8519 | 0,6204 | 0,1785 | 0,0856 | 0,0397 | 0,0591 | 0,1854 | 0,2030 | 0,1111 | 0,4227 |
| Aβ8-40 | 0,0028 | 0,0013 | <,0001 | 0,0144 | 0,0067 | 0,7790 | 0,3460 | 0,9400 | 0,0096 | 0,0251 | 0,5077 | 0,6483 | 0,5605 | 0,1575 | 0,7031 | 0,0706 |
| Aβ7-40 | <,0001 | <,0001 | 0,0144 | <,0001 | 0,8346 | 0,0526 | 0,3581 | 0,3490 | 0,2323 | 0,1108 | 0,0109 | 0,0192 | 0,3838 | 0,2258 | 0,0711 | 0,1274 |
| Aβ5-40 | 0,1109 | 0,6854 | 0,0067 | 0,8346 | <,0001 | 0,1437 | 0,0802 | 0,0878 | <,0001 | 0,0079 | 0,7254 | 0,8626 | 0,4718 | 0,2978 | 0,0870 | 0,7157 |
| Aβ4-40 | 0,0606 | 0,0548 | 0,7790 | 0,0526 | 0,1437 | <,0001 | 0,1013 | 0,7327 | 0,1758 | 0,1071 | 0,1658 | 0,2539 | 0,0009 | 0,8742 | 0,7120 | 0,0996 |
| Aβ3pE-40 | 0,5924 | 0,8519 | 0,3460 | 0,3581 | 0,0802 | 0,1013 | <,0001 | <,0001 | 0,9851 | 0,4884 | 0,0004 | 0,0158 | 0,3745 | 0,0437 | 0,8868 | 0,9320 |
| Aβ3-40 | 0,5530 | 0,6204 | 0,9400 | 0,3490 | 0,0878 | 0,7327 | <,0001 | <,0001 | 0,7756 | 0,0360 | 0,0181 | 0,3825 | 0,2135 | <,0001 | 0,4989 | 0,5527 |
| Aβ2-40 | 0,0278 | 0,1785 | 0,0096 | 0,2323 | <,0001 | 0,1758 | 0,9851 | 0,7756 | <,0001 | 0,0735 | 0,4680 | 0,2740 | 0,7466 | 0,7106 | 0,0395 | 0,8926 |
| Aβ1-40 | 0,0348 | 0,0856 | 0,0251 | 0,1108 | 0,0079 | 0,1071 | 0,4884 | 0,0360 | 0,0735 | <,0001 | 0,0477 | 0,0087 | 0,0032 | 0,1442 | 0,2800 | 0,7867 |
| Aβ11pE-42 | 0,2122 | 0,0397 | 0,5077 | 0,0109 | 0,7254 | 0,1658 | 0,0004 | 0,0181 | 0,4680 | 0,0477 | <,0001 | <,0001 | 0,1170 | 0,1113 | 0,3112 | 0,7248 |
| Aβ11-42 | 0,1600 | 0,0591 | 0,6483 | 0,0192 | 0,8626 | 0,2539 | 0,0158 | 0,3825 | 0,2740 | 0,0087 | <,0001 | <,0001 | 0,0236 | 0,9568 | 0,0296 | 0,3055 |
| Aβ5-42 | 0,2937 | 0,1854 | 0,5605 | 0,3838 | 0,4718 | 0,0009 | 0,3745 | 0,2135 | 0,7466 | 0,0032 | 0,1170 | 0,0236 | <,0001 | 0,0580 | 0,9673 | 0,5465 |
| Aβ4-42 | 0,9334 | 0,2030 | 0,1575 | 0,2258 | 0,2978 | 0,8742 | 0,0437 | <,0001 | 0,7106 | 0,1442 | 0,1113 | 0,9568 | 0,0580 | <,0001 | 0,6924 | 0,0273 |
| Aβ3pE-42 | 0,0186 | 0,1111 | 0,7031 | 0,0711 | 0,0870 | 0,7120 | 0,8868 | 0,4989 | 0,0395 | 0,2800 | 0,3112 | 0,0296 | 0,9673 | 0,6924 | <,0001 | 0,1322 |
| Aβ1-42 | 0,1831 | 0,4227 | 0,0706 | 0,1274 | 0,7157 | 0,0996 | 0,9320 | 0,5527 | 0,8926 | 0,7867 | 0,7248 | 0,3055 | 0,5465 | 0,0273 | 0,1322 | <,0001 |

### Scatterplot Matrix

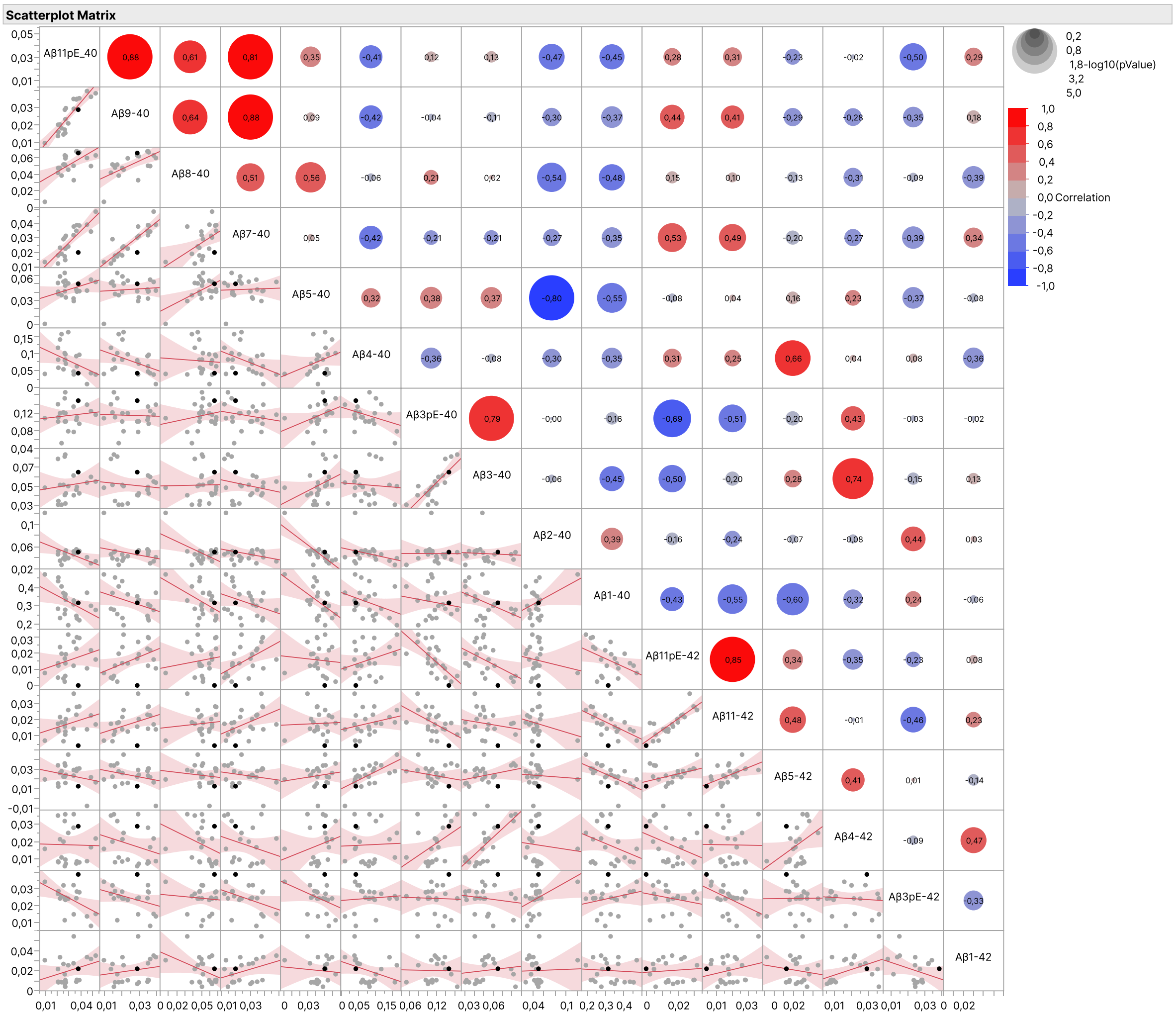


**Figure S17.**

Correlation analysis of amyloid peptides in CAA, Correlation matrix of all amyloid peptides indicating both correlation plots, significance (p value) and correlation $R^2$.
