# Supplementary material for: Chemical imaging delineates Aβ plaque polymorphism across the Alzheimer’s disease spectrum
Source: Nat Commun. 2025 Apr 24;16:3889. doi: 10.1038/s41467-025-59085-7 (PMC12022071; doi:10.1038/s41467-025-59085-7)
Supplement: Supplementary file 2 — Reporting Summary [file 41467_2025_59085_MOESM2_ESM.pdf]

nature portfolio

Corresponding author(s): Jörg Hanrieder

Last updated by author(s): Mar 25, 2025

Reporting Summary

Nature Portfolio wishes to improve the reproducibility of the work that we publish. This form provides structure for consistency and transparency in reporting. For further information on Nature Portfolio policies, see our Editorial Policies and the Editorial Policy Checklist.

Statistics

For all statistical analyses, confirm that the following items are present in the figure legend, table legend, main text, or Methods section.

n/a	Confirmed	
☐	☒	The exact sample size (*n*) for each experimental group/condition, given as a discrete number and unit of measurement
☐	☒	A statement on whether measurements were taken from distinct samples or whether the same sample was measured repeatedly
☐	☒	The statistical test(s) used AND whether they are one- or two-sided<br>*Only common tests should be described solely by name; describe more complex techniques in the Methods section.*
☐	☒	A description of all covariates tested
☐	☒	A description of any assumptions or corrections, such as tests of normality and adjustment for multiple comparisons
☐	☒	A full description of the statistical parameters including central tendency (e.g. means) or other basic estimates (e.g. regression coefficient) AND variation (e.g. standard deviation) or associated estimates of uncertainty (e.g. confidence intervals)
☐	☒	For null hypothesis testing, the test statistic (e.g. *F*, *t*, *r*) with confidence intervals, effect sizes, degrees of freedom and *P* value noted<br>*Give P values as exact values whenever suitable.*
☒	☐	For Bayesian analysis, information on the choice of priors and Markov chain Monte Carlo settings
☐	☒	For hierarchical and complex designs, identification of the appropriate level for tests and full reporting of outcomes
☐	☒	Estimates of effect sizes (e.g. Cohen's *d*, Pearson's *r*), indicating how they were calculated

Our web collection on statistics for biologists contains articles on many of the points above.

Software and code

Policy information about availability of computer code

Data collection	MALDI-MSI experiments were performed on a rapifleX Tissuetyper MALDI-TOF/TOF instrument (Bruker Daltonics) using the FlexImaging and FlexControl (v5.0, Bruker Daltonics) software. Light microscopy was performed on automatic widefield microscope (Axio Observer Z1, Zeiss, Germany).
Data analysis	Data analysis was performed using FlexImaging (v5.1, Bruker Daltonics), Origin (version 8.1 OriginLab, Northampton, MA), Prism (v.9, GraphPad, San Diego, CA, USA) and Metaboanalyst 5.0, FIJIImageJ

For manuscripts utilizing custom algorithms or software that are central to the research but not yet described in published literature, software must be made available to editors and reviewers. We strongly encourage code deposition in a community repository (e.g. GitHub). See the Nature Portfolio guidelines for submitting code & software for further information.

Data

Policy information about availability of data

All manuscripts must include a data availability statement. This statement should provide the following information, where applicable:

- Accession codes, unique identifiers, or web links for publicly available datasets
- A description of any restrictions on data availability
- For clinical datasets or third party data, please ensure that the statement adheres to our policy

All MALDI plaque ROI mass spectral data are available at: https://maciejdulewiczgu.shinyapps.io/Check_my_SpectRa/ . All relevant peptide LCMSMS data are available at the PRIDE repository. Source data of all box- and barplots are provided with the manuscript.

Research involving human participants, their data, or biological material

Policy information about studies with human participants or human data. See also policy information about sex, gender (identity/presentation), and sexual orientation and race, ethnicity and racism.

Reporting on sex and gender	Our study includes data from 7 males and 16 females. However, sex was not treated as a biological variable.
Reporting on race, ethnicity, or other socially relevant groupings	We did not consider social variables in this study.
Population characteristics	Frozen brain tissue samples were obtained from temporal cortex of individuals who had been clinically and pathologically diagnosed with sporadic AD (sAD, n=9) and autosomal dominantly inherited familial AD (fAD, n=6). All cases were obtained through the brain donation program of the Queen Square Brain Bank for Neurological Disorders (QSBB), Department of Clinical and Movement Neurosciences, UCL Queen Square Institute of Neurology. The standard diagnostic criteria were used for the neuropathological diagnosis of AD (Braak and Braak, 1991; Thal et al., 2002; Montine et al., 2012; Thal et al., 2015).
Recruitment	n/a
Ethics oversight	Ethical approval for the study was obtained from the Local Research Ethics Committee of the National Hospital for Neurology and Neurosurgery, as well as the Ethics Review Board at the University of Gothenburg (Gothenburg, 04/16/2015; DNr 012-15). The Queen Square Brain Bank (QSBB) operates under ethical approval that permits the collection, storage, and use of donated tissue and associated clinical data for research purposes. This includes the use of de-identified data in research projects.

Note that full information on the approval of the study protocol must also be provided in the manuscript.

Field-specific reporting

Please select the one below that is the best fit for your research. If you are not sure, read the appropriate sections before making your selection.

[x] Life sciences [ ] Behavioural & social sciences [ ] Ecological, evolutionary & environmental sciences

For a reference copy of the document with all sections, see nature.com/documents/nr-reporting-summary-flat.pdf

Life sciences study design

All studies must disclose on these points even when the disclosure is negative.

Sample size	Brain tissue samples from 23 individuals diagnosed post mortem with Alzheimer's disease were included in the study.
Data exclusions	No data were excluded
Replication	The number of technical replicate analyses (tissue sections) per patients was N=2. Intra and inter patient variation was determined within the respective groups and across the technical replicates. Further, the specific technical variance of the MALDI MSI technique was estimated by MSI analysis of three sequential sections from one patient. No statistical method was used to predetermine sample size. No data were excluded from the analyses.
Randomization	The experiments were randomized .
Blinding	The investigators performing MSI and IHC data acquisition and data processing were blinded to allocation during experiments and outcome assessment.

Reporting for specific materials, systems and methods

We require information from authors about some types of materials, experimental systems and methods used in many studies. Here, indicate whether each material, system or method listed is relevant to your study. If you are not sure if a list item applies to your research, read the appropriate section before selecting a response.

Materials & experimental systems

n/a	Involved in the study
☐	☒ Antibodies
☒	☐ Eukaryotic cell lines
☒	☐ Palaeontology and archaeology
☒	☐ Animals and other organisms
☒	☐ Clinical data
☐	☐ Dual use research of concern
☒	☐ Plants

Methods

n/a	Involved in the study
☒	☐ ChIP-seq
☒	☐ Flow cytometry
☒	☐ MRI-based neuroimaging

Antibodies

Antibodies used

PHF-1 (Courtesy of Dr. Peter Davies, Feinstein Institute for Medical Research), RTN3 (Millipore sigma, Catalog # ABN1723), 6E10 (anti-amyloidβ 1-16) (BioLegend, Catalog # 803003), Alexafluor594 - Thermofischer Catalog # A32740 and/or Alexafluor647 - Thermofischer Catalog # A32728

Validation

Validation of primary antibody as summarized from manufacturers protocols:

anti-amyloidβ 1-16, 6E10, https://www.biolegend.com/en-gb/products/purified-anti-beta-amyloid-1-16-antibody-11228?GroupID=BLG15648

MMouse anti-Aβ (6E10, #SIG-39320)
Link: https://www.biolegend.com/en-gb/products/purified-anti-beta-amyloid-1-16-antibody-11228?GroupID=BLG15648

Datasheet: https://d1spbj2x7qk4bg.cloudfront.net/en-gb/products/purified-anti-beta-amyloid-1-16-antibody-11228?displayInline=true&filename=Purified%20anti-%CE%B2-Amyloid,%201-16%20%20Antibody.pdf&leftRightMargin=15&pdf=true&topBottomMargin=15&v=20241208073714

Validation Description: Western blot of purified anti-β-amyloid, 1-16 antibody (clone 6E10)

Citations (383):
1. Abud EM et al. 2017. Neuron. 94(2):278-293 . PubMed
2. Wang X, et al. 2019. Cell Res. 29:787. PubMed
3. Eede P, et al. 2020. EMBO Rep. 21:e48530. PubMed
4. Sogorb-Esteve A, et al. 2018. Mol Neurobiol. 55:5047. PubMed
5. Turnbull MT, et al. 2018. Front Mol Neurosci. 11:51. PubMed
6. Singh N, et al. 2022. Sci Adv. 8:eabo1286. PubMed
7. Ye Q, et al. 2022. Neurobiol Dis. 172:105820. PubMed
8. Guo T, et al. 2022. J Neurosci. . PubMed
9. Vasilopoulou MA, et al. 2022. Redox Biol. 56:102462. PubMed
10. Yu H, et al. 2023. Alzheimers Dement. 19:2365. PubMed
11. Williams D, et al. 2023. Sci Rep. 13:2337. PubMed
12. Rimal S, et al. 2023. EMBO Rep. 24:e55548. PubMed

RTN3, https://www.emdmillipore.com/US/en/product/Anti-RTN3-R458,MM_NF-ABN1723

Plants

Seed stocks

Report on the source of all seed stocks or other plant material used. If applicable, state the seed stock centre and catalogue number. If plant specimens were collected from the field, describe the collection location, date and sampling procedures.

Novel plant genotypes

Describe the methods by which all novel plant genotypes were produced. This includes those generated by transgenic approaches, gene editing, chemical/radiation-based mutagenesis and hybridization. For transgenic lines, describe the transformation method, the number of independent lines analyzed and the generation upon which experiments were performed. For gene-edited lines, describe the editor used, the endogenous sequence targeted for editing, the targeting guide RNA sequence (if applicable) and how the editor was applied.

Authentication

Describe any authentication procedures for each seed stock used or novel genotype generated. Describe any experiments used to assess the effect of a mutation and, where applicable, how potential secondary effects (e.g. second site T-DNA insertions, mosiacism, off-target gene editing) were examined.
